# A Novel Synthetic Tag Induces Palmitoylation and Directs the Subcellular Localization of Target Proteins

**DOI:** 10.3390/biom15081076

**Published:** 2025-07-25

**Authors:** Jun Ka, Gwanyeob Lee, Seunghyun Han, Haekwan Jeong, Suk-Won Jin

**Affiliations:** 1 School of Life Sciences, Gwangju Institute of Science and Technology (GIST), Gwangju 61005, Republic of Koreaigy8433@gmail.com (G.L.);; 2 Astron Bio, Inc., Room 629, 29, Jukjeon-ro, Giheung-gu, Yongin-si 16898, Gyeonggi-do, Republic of Korea; 3 Department of Biological Sciences, Korea Advanced Institute of Science and Technology, Daejeon 34141, Republic of Korea

**Keywords:** palmitoylation, acylation, trafficking, extracellular vesicles, sequence tag

## Abstract

Proper subcellular localization is essential to exert the designated function of a protein, not only for endogenous proteins but also transgene-encoded proteins. Post-translational modification is a frequently used method to regulate the subcellular localization of a specific protein. While there are a number of tags that are widely used to direct the target protein to a specific location within a cell, these tags often fail to emulate the dynamics of protein trafficking, necessitating an alternative approach to the direct subcellular localization of transgene-encoded proteins. Here, we report the development of a new synthetic polypeptide protein tag comprised of ten amino acids, which promotes membrane localization of a target protein. This short synthetic peptide tag, named “Palmito-Tag”, induces ectopic palmitoylation on the cysteine residue within the tag, thereby promoting membrane localization of the target proteins without affecting their innate function. We show that the target proteins with the Palmito-Tag are incorporated into the membranous organelles within the cells, including the endosomes, as well as extracellular vesicles. Given the reversible nature of palmitoylation, the Palmito-Tag may allow us to shift the subcellular localization of the target protein in a context-dependent manner. With the advent of therapeutic applications of exosomes and other extracellular vesicles, we believe that the ability to reversibly modify a target protein and direct its deposition to the specific subcellular milieu will help us explore more effective venues to harness the potential of extracellular vesicle-based therapies.

## 1. Introduction

Eukaryotic cells possess diverse subcellular organelles, which are highly complex and compartmentalized structures containing various membranous structures. Spatial separation of these subcellular organelles provides additional layers of regulation for the synthesis, modification, and secretion of proteins [1,2,3]. More importantly, such subcellular compartmentalization allows a single protein to exert distinct functions based on its location within the cell. Indeed, a significant number of proteins, of which functions are essential for cellular physiology, are known to occupy multiple subcellular locations in a context-dependent manner and display dynamic subcellular trafficking behaviors [4]. Similar to endogenous proteins, transgene-encoded proteins also need to occupy specific subcellular locations to exert designated functions. While various tags can direct these proteins to a specific subcellular milieu [5], these are not efficient at emulating dynamic trafficking.

Nascent proteins undergo diverse modifications by being decorated with diverse moieties such as acetylglucosamine, polysaccharides, and free fatty acids [6], which help them to occupy a specific subcellular location. While it is thought that post-translational modifications are irreversible and permanently alter the structure and/or hydrophobicity of the target proteins [7], certain modifications such as phosphorylation, palmitoylation, and ubiquitination appear to be reversible, allowing context-dependent modulations of the target protein activity. These reversible modifications can also render dynamic trafficking and change the subcellular localization of the target proteins [6,8].

Palmitoylation is a type of acylation that adds palmitates to a cysteine residue of the target protein. Palmitoylation could alter the trafficking and subcellular localization of both membrane and cytosolic proteins by increasing their hydrophobicity [6]. The unique feature of palmitoylation is that it is a reversible process, which sets it apart from other forms of acylation such as prenylation and myristoylation [9]. Palmitoylation is regulated by distinct sets of enzymes: palmitoyl acyltransferases (PATs), also known as DHHCs [10,11], and acyl-protein thioesterase (APTs) or ABHD proteins. PATs add palmitate to the protein, while APTs and ABHD remove the palmitate from the protein [9]. In order to function, PATs/DHHCs recognize a stretch of specific amino acids within themselves known as the DHHC sequence and undergo auto-palmitoylation [11,12].

It has been proposed that PATs/DHHCs recognize a short, highly conserved stretch of amino acids, Asp-His-His-Cys, within themselves to undergo auto-palmitoylation. This sequence, known as the DHHC motif, appears to be crucial for the activation of PATs/DHHCs. However, beyond this conserved sequence, the roles of adjacent amino acids, which may influence the interaction between PATs/DHHCs and their targets, remain poorly understood. Recently, a consensus target sequence for auto-palmitoylation was identified in two PATs/DHHCs. DHHC13 and DHHC17 appear to recognize an internal amino acid sequence, [VIAP] [VIT] XXQP, to undergo auto-palmitoylation [12]. However, whether this sequence can serve as a consensus motif for auto-palmitoylation still requires further validation. Additionally, it is unclear whether this consensus sequence could function as a recognition motif for exogenous targets, given that PATs/DHHCs can also bind to their targets through alternative sequences, such as PDZ-binding motifs, SH3 domains, and ankyrin-repeat domains [11,12].

As a reversible process, protein palmitoylation promotes dynamic changes in subcellular localization for shuttling proteins. For instance, palmitoylation is known to dictate the subcellular localization of Ras [13] and STAT3 [14] to modulate signaling and differentiation. Here, we developed new synthetic short polypeptide protein tags, the ‘Palmito-Tags’, which promote palmitoylation of target proteins. We demonstrated that these Palmito-Tags can direct the subcellular localization of target proteins by facilitating palmitoylation of the cysteine residue within the tag. As the Palmito-Tag appears to undergo palmitoylation, which is a reversible process, it could apply to emulating dynamic protein trafficking within the cell and allow us to expand our current toolkit for protein design.

## 2. Materials and Methods

### 2.1. Cell Culture

Human embryonic kidney 293T cells (CRL-3216, ATCC, Manassas, VA, USA) were cultured in high glucose Dulbecco’s modified Eagle’s medium (DMEM; 10-013-CV, Corning, Manassas, VA, USA) containing 10% fetal bovine serum (FBS; 35-015-CV, Corning) or Exosome-Depleted FBS (A2720803, Gibco, Gaithersburg, MD, USA) and 1% penicillin-streptomycin (SV30010, Cytiva, Amersham, UK). Human umbilical vein endothelial cells (HUVECs; C0035C, Gibco) were cultured in Endothelial Cell Growth Medium MV2 supplemented with SupplementMIX MV2 (C-22022, PromoCell, Heidelberg, Germany) and 1% penicillin-streptomycin (SV30010, Cytiva). Cells were maintained at 37 °C in 5% CO_2_ in the incubator (Thermo Fisher Scientific, Waltham, MA, USA).

### 2.2. Constructs and Transfection

All PCR primers used in this study are listed in Table S1. Palmito-Tag sequences were genetically fused to the NH_2_ terminus of cargo proteins including EGFP, nanoluciferase, β-galactosidase and Cre recombinase, by PCR. To generate the pcDNA3.1-PalmitoTag-cargo constructs, sequences encoding full-length Cargo proteins were amplified by PCR from pCCL-MCS-miniCMV-EGFP (plasmid: 134984, Addgene, Watertown, MA, USA), pUAS-NanoLuc (plasmid: 87696, Addgene), pShuttle-Cre-HA (plasmid: 16583, Addgene), and pCMX-β-GAL (kindly provided by Mi-Ryoung Song) vectors. Amplified PCR fragments were inserted at HindIII and XhoI sites of the pcDNA3.1-ShhN (plasmid: 37680, Addgene) vector. Mcherry-CD9-10 (plasmid: 55013, Addgene) vector was intactly used to label EVs and membranous organelles. Cells were transfected with DNA constructs using polyethyleneimine (23966-1, Polysciences, Warrington, PA, USA) to express genes of interest. Transfected cells were selected using conditioned medium with G-418 (Roche, Basel, Switzerland) and hygromycin B (H7772, Sigma-Aldrich, St. Louis, MO, USA) or fractioned by a FACS Calibur machine (BD Biosciences, Franklin Lakes, NJ, USA).

### 2.3. EV Production and Isolation

EVs were isolated by sequential ultracentrifugation. Conditioned medium was collected from HEK293T cells incubated with culture medium supplemented with 10% exosome-depleted FBS for 72 h. Collected culture medium was centrifuged at 300× *g* for 10 min and 2000× *g* at 4 °C to remove cells and dead cells and filtered through a 0.2 μm or 0.8 μm syringe filter (Corning). Filtered medium was centrifuged at 10,000× *g* for 40 min and 100,000× *g* for 70 min at 4 °C. Pellets were washed with ice-cold Dulbecco’s phosphate-buffered saline (DPBS; LB001-02, Welgene, Seoul, Republic of Korea) to remove contaminating proteins and centrifuged at 100,000× *g* for 70 min at 4 °C. EV pellets were resuspended with ice-cold DPBS with protease and phosphate inhibitor cocktail (78442, Thermo Fisher Scientific). The size and number of isolated EVs were measured by a Zetaview nanoparticle tracking analyzer (Particle Metrix, Inning am Ammersee, Germany).

### 2.4. Detection of Palmitoylated Proteins

HEK293T cells were transfected using EGFP with the Palmito-tag and harvested 30 h after transfection. Palmitoylated proteins were captured by using resin-assisted capture of S-acylated proteins according to the manufacturer’s instructions (K010-311, Badrilla, Leeds, UK). For the click reactions, HEK293T cells were treated with 100 uM of azido-palmitic acid for 6 h (1346-100, Click chemistry tools) before harvest. Click-reaction was performed to label palmitoylated proteins using biotin-alkyne (B10185, Thermo Fisher Scientific) according to manufacturer’s instructions (1262, Click chemistry tools). Labeled proteins were captured using streptavidin agarose beads (SA10004, Thermo Fisher Scientific) and used for western blot.

### 2.5. Immunoblotting

Collected cells or isolated EVs were lysed with RIPA lysis and extraction buffer (89901, Thermo Fisher Scientific), resolved by 10% SDS-PAGE, and transferred onto PVDF membranes. The membranes were blocked with 5% bovine serum albumin (BSA; LPS solution, Dajeon, Republic of Korea), immunoblotted with mouse monoclonal GFP antibody (1:500, MA5-15256, Invitrogen, Waltham, MA, USA), rabbit polyclonal mCherry antibody (1:500, PA5-34974, Invitrogen), mouse monoclonal Alix antibody (1:100, SC53540, Santa Cruz Biotechnology, Dallas, TX, USA), mouse monoclonal nanoluciferase antibody (1:200, MAB10026, R&D Systems, Minneapolis, MN, USA), rabbit monoclonal Cre antibody (1:1000, 15036, Cell Signaling Technology, Danvers, MA, USA), rabbit polyclonal β-galactosidase antibody (1:500, A-11132, Invitrogen), and mouse monoclonal GAPDH antibody (1:1000, MA5-15738, Invitrogen). Afterward, the immunoblots were incubated with horseradish peroxidase-conjugated secondary anti-mouse IgG (1:5000, 62-6520, Invitrogen) and anti-rabbit IgG (1:5000, ab97051, Abcam, Cambridge, UK) antibodies. Signal was detected with enhanced chemiluminescent Select Western Blotting detection reagent (RPN2235, Cytiva, Marlborough, MA, USA).

### 2.6. Confocal Microscopy for Cell Imaging

To analyze the change of subcellular localization of Palmito-Tag proteins and CD9-mcherry, transfected cells were imaged using an FV3000RS confocal microscope (Olympus, Shinjuku, Tokyo, Japan) with 488 nm and 561 nm laser stimulation. Cells on a 35-mm glass bottom confocal dish (200350, SPL Life Sciences, Pocheon, Republic of Korea) were fixed using 4% paraformaldehyde solution for 15 min at RT and mounted using Fluoroshield (Sigma-Aldrich). Anti-nanoluciferase (1:100) and Alexa Fluor 488 donkey secondary antibodies (Invitrogen) were used for immunofluorescence of nanoluciferase. To image subcellular localization of nanoluciferase, the fixed cells were permeabilized using PBST (0.1% Triton X-100 containing PBS) and incubated overnight at 4 °C in PBST with primary antibody. Then, the cells were incubated with Alexa Fluor 488 secondary antibody and mounted. The fluorescence was quantitated using FV31S-SW (Olympus) and IMARIS 9.3 software (Andor Technology, Belfast, UK).

### 2.7. EV Uptake Analysis

To assess the uptake of cargo-loading EVs by recipient cells, 6 × 104 HUVEC cells were seeded in each well of a 4-well cell culture slide (30504, SPL Life Sciences). After a day, isolated EV particles from EV donors were incubated with HUVEC cells for 6 h, 1.3 × 108 particles per well. EV-containing medium was then removed, and recipient cells were washed with PBS 5 times for 20 min. Then, cells were fixed using 4% paraformaldehyde followed by permeabilization using PBST. The cell nuclei and actin filaments were stained using Hoechst 33,342 and Alexa Fluor 647 Phalloidin (Thermo Fisher Scientific). The chamber of the cell culture slide was removed, followed by mounting with Fluoroshield. Cells were imaged using an FV3000RS confocal microscope, and internalization of EVs was assessed by Z-stack imaging.

### 2.8. Luciferase Activity Assay

For assessment of the function of Palmito-Tag-Nanoluciferase, cells or isolated EVs were rinsed in PBS and lysed with Lysis Reagent (Promega, Madison, WI, USA). A total volume of 20-μL samples per well was loaded into a white 96-well plate (Thermo Fisher Scientific). Then, 100 μL of Luciferase Assay Reagent (Promega) was added and mixed. For the live cell luciferase assay, the Nano-Glo Live Cell Luciferase Assay System (Promega) was used following the manufacturer’s protocol. EV recipient cells were seeded into a white 96-well plate. Isolated EVs were incubated with recipient cells for 3 h and washed 5 times with exosome-depleted medium. Luciferase activity measurements were performed using a plate reader (Flexstation3, Molecular Devices, San Jose, CA, USA).

### 2.9. Statistical Analyses

Statistical analyses were conducted using GraphPad Prism 6 software. Statistical significance was assessed using a Student’s unpaired *t*-test. One-way analysis to compare groups was performed using Tukey’s test. Data are presented as mean (SD).

## 3. Results

### 3.1. Design and Optimization of the Palmito-Tag

**Figure 1:**
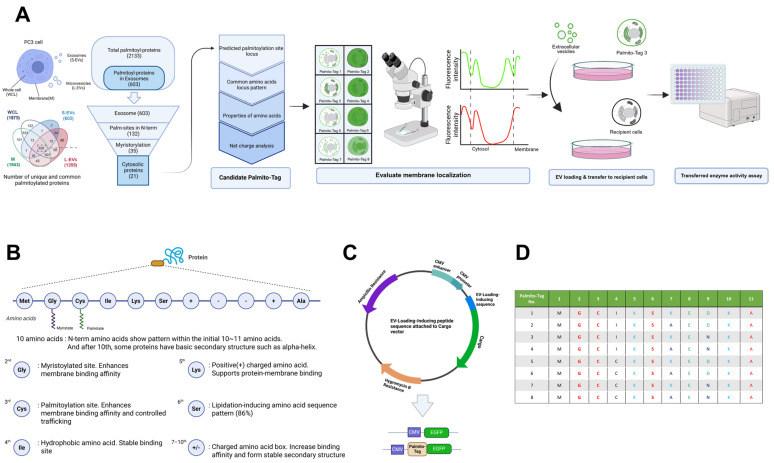
Deducing the consensus sequence for palmitoylation and design of the Palmito-Tag. (**A**) Schematic diagram for the experiment. Brute-force screening and analyses of previously reported palmitoylated proteins. Among the 2133 known palmitoylated proteins, 603 proteins that are localized to extracellular vesicles were selected for further analysis. Among those, 132 proteins that undergo palmitoylation at their N-terminus were selected, and then 21 cytosolic proteins that also undergo myristoylation were selected for further sequence analysis. Membrane proteins were excluded, as they contain a signal peptide sequence at their N-terminus. (**B**) Deduced consensus sequence for palmitoylation. The prevalence of amino acids occupying each position within the sequence was compared among selected palmitoylated proteins. Methionine was added at the 5′– end of the sequence as the Palmito-Tag was designed to fuse at the N-terminus of the target protein, and scissors indicate cleavage of N-terminal Met. (**C**) Schematic illustration of the construct designed for Palmito-Tag subcloning. (**D**) To determine the optimal amino acid for each position, eight variants of the Palmito-Tag were generated.

In order to generate a synthetic sequence that could induce palmitoylation, we have analyzed the sequences of proteins from public palmitoylated proteomics data obtained from prostate cancer cells that have been reported to undergo palmitoylation for localization into extracellular vesicles by Mariscal et al. [15]. Previously, among 2133 of the total palmitoylated proteins, 2011 proteins occupy specific subcellular milieus, with 1355 within microvesicles and exosomes and 1843 within the membranous organelle, have been reported (Figure 1A) [15]. To deduce the consensus amino acid sequence, which can serve as a target for palmitoylation, we examined the predicted palmitoylation sequence in the palmitoylated proteins localized to the secretory vesicles. As 603 palmitoylated proteins have been proposed to be localized to the exosomes [15], we first selected those proteins for further analyses. Among these, 132 proteins, or 21.9% of the palmitoylated proteins which are localized to the secretory vesicles, are predicted to undergo palmitoylation at their N-terminus. Among these, we selected those that also undergo myristoylation, an irreversible post-translational modification of N-terminal glycine, as it has been reported to facilitate subsequent palmitoylation in certain proteins [7,16,17,18]. Consistent with this idea, we found that 26% of palmitoylated proteins in their N-term undergo myristoylation. Next, we excluded integral membrane proteins and identified 21 proteins that are known to localize within the cytosol yet undergo palmitoylation. Based on their sequence encompassing the putative palmitoylation site, we deduced the potential consensus sequence, which can induce palmitoylation in cytosolic proteins. We further refined the target sequence by net charge analysis and designed eight candidate sequences for the Palmito-Tag, all of which contain a consensus sequence of the Met-Gly-Cys stretch in the N-terminus of the sequence, followed by a hydrophobic amino acid in the fourth position and a positively charged amino acid in the fifth position, respectively, and Ser in the sixth position, which appeared to be an important residue among the lipidated proteins [17]. We added four additional amino acids at the C-terminus of the sequence to stabilize the secondary structures of the Palmito-Tag (Figure 1B). The eight candidate sequences were subcloned into the upstream of EGFP for further analyses (Figure 1C,D).

### 3.2. Subcellular Localization of EGFP Was Altered by the Palmito-Tag

**Figure 2:**
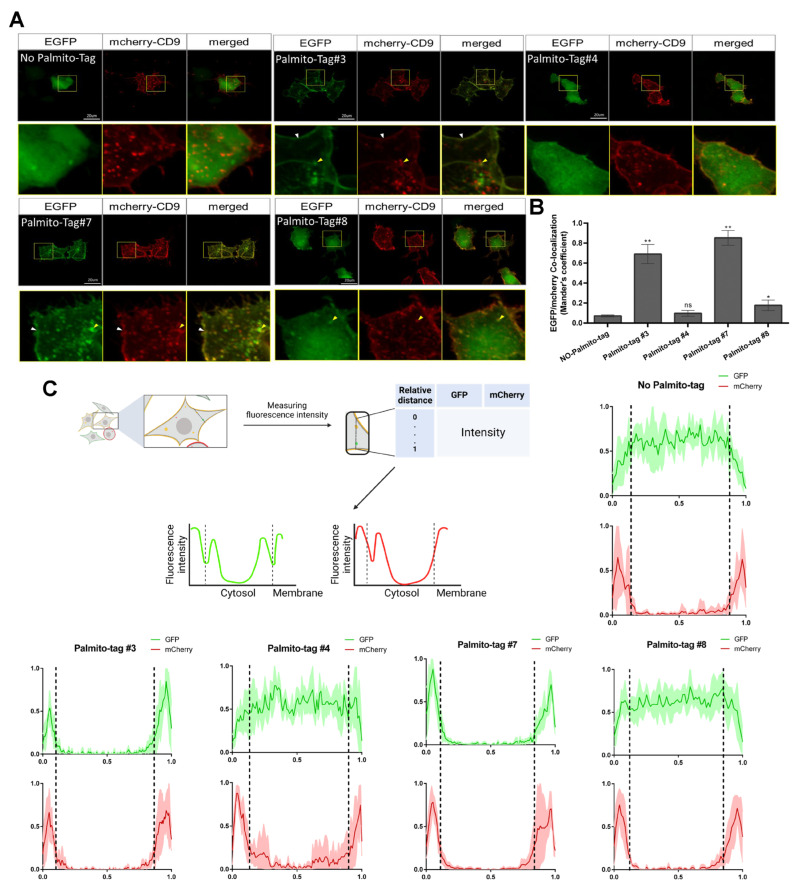
Palmito-Tag can direct the target protein to the membranous structure. (**A**) Subcellular localization of the GFP tagged with Palmito-Tags 3, 4, 7, or 8, along with the non-tagged GFP. Compared to the control GFP, which showed ubiquitous distribution within the cells, those tagged with either Palmito-Tags 3 or 7 were preferentially localized to the membranous subcellular structure, including the plasma membrane, which is characterized by co-localization with mCherry-CD9, while those tagged with either Palmito-Tags 4 or 8 were localized to both the membranous structure and cytosolic compartments. Yellow arrowheads point to co-localization of GFP and mCherry-CD9 in intracellular membranous structures, while white arrowheads point to co-localization of GFP and mCherry-CD9 at the plasma membrane (colors: EGFP-Palmito-Tag (green) and mCherry-CD9 (red); scale bar = 20 μM). (**B**) Quantification of the percentage of GFP co-localized with mCherry-CD9. The data is presented as the mean (SD). * indicates *p* < 0.05, and ** indicates *p* < 0.01. (**C**) XY plot of fluorescence intensity of EGFP and mCherry-CD9. A diagram shows the measuring method used to plot graphs. The X-axis indicates the relative distance from the measuring start point, while the Y-axis represents the intensity of each fluorescence.

We then examined the subcellular localization of EGFP tagged with different Palmito-Tags to assess the ability of individual Palmito-Tags to direct subcellular localization of the target protein. Compared to the control EGFP, which does not have any extra N-terminus tag, the EGFP which was fused with Palmito-Tag showed membrane localization to varying degrees (Figure 2A and Figure S1). For instance, EGFP with Palmito-Tags 1, 3, and 7 shows complete overlapping localization with mCherry-CD9, which has previously been shown to localize to the plasma membrane [19] (Figure 2A and Figure S1), with over 60% of EGFP with Palmito-Tag 3 being co-localized with mCherry-CD9. EGFP with Palmito-Tags 4 and 8, in comparison, shows partial overlap with mCherry-CD9 with a significant portion being within the cytoplasm, as only 9.7% of EGFP appears to be co-localized with mCherry-CD9 (Figure 2A–C and Figure S1). We found that EGFP with Palmito-Tags 1, 3, and 7 was preferentially localized to the plasma membrane compared to EGFP with other Palmito-Tags (Figure 2A,C). To select the optimal sequence for further analyses, we compared the expression level as well as the subcellular distribution of EGFP tagged with these three candidates. We found that EGFP tagged with either Palmito-Tags 3 or 7 were more robustly expressed than EGFP tagged with Palmito-Tag 1 (Figure 2A and Figure S1). In addition, EGFP tagged with Palmito-Tag 3 appeared to be more selectively localized to the membranous structure than the Palmito-Tag 7-containing EGFP. Therefore, we utilized Palmito-Tag3 for further analysis. Taken together, our data illustrate that a subtle sequence variation within the Palmito-Tag could result in a distinct subcellular localization of a target protein.

**Figure 3:**
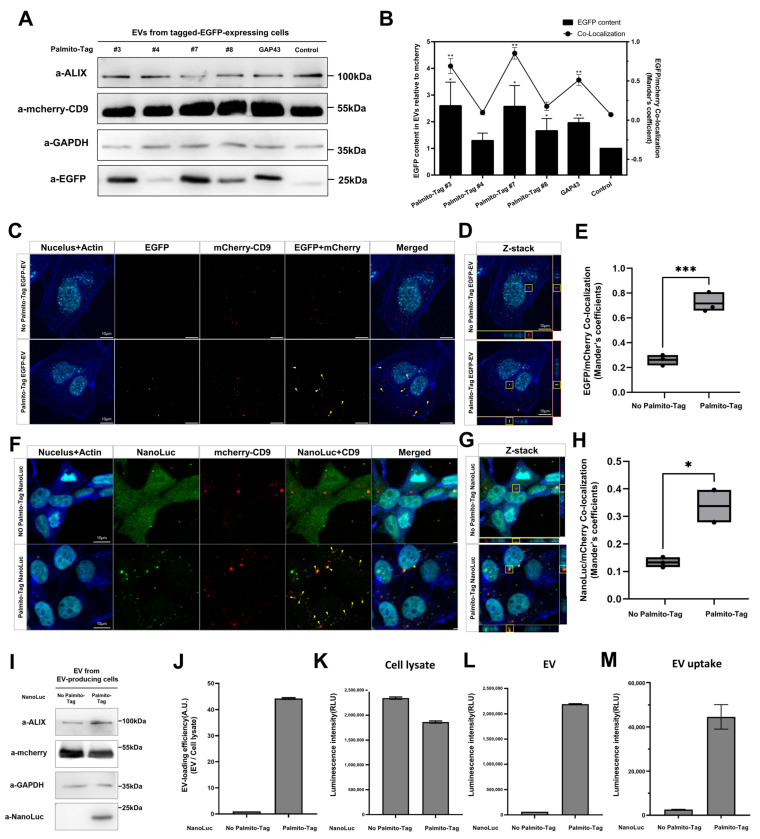
Palmito-Tag leads to target protein deposition within the extracellular vesicles. (**A**) Western blots of isolated extracellular vesicles show conditioned media from the cell culture expressing the GFP fused with the Palmito-Tag contained a significant amount of EGFP within the extracellular vesicles. EGFP fused with the GAP43 sequence at the N-terminus was used as a positive control. Palmito-Tags 3 and 7 appear to be more effective than other Palmito-Tags in depositing EGFP into the extracellular vesicles (Original images can be found in Supplementary Materials). (**B**) Quantification of EGFP content in extracellular vesicles is overlayed with the percentage of EGFP and mCherry-CD9 co-localization. The amount of EGFP loaded on the extracellular vesicles appears to have a positive correlation to the percentage of EGFP co-localized with mCherry. Data are presented as mean (SD). * indicates *p* < 0.05, and ** indicates *p* < 0.01. (**C**,**D**) Extracellular vesicles harvested from HEK293T cells transfected with EGFP with and without the Palmito-Tag were treated to HUVECs without transfection of both EGFP and mCherry-CD9. (**C**,**D**) Confocal images of extracellular vesicle recipient cells (colors: EGFP-Palmito-Tag (green), DAPI (cyan), F-actin (blue), and mCherry-CD9 (red); scale bar = 10 μM). (**E**) Quantification of EGFP and mCherry-CD9 co-localization in recipient cells. *** indicates *p* < 0.001 (**F**,**G**) Nanoluciferase (NanoLuc) with or without Palmito-Tag #3 was expressed with mCherry-CD9 in HEK293T cells. (**F**,**G**) Confocal images of transfected HEK293T cells using NanoLuc with Palmito-Tag and mCherry-CD9 (colors: NanoLuc-Palmito-Tag (green), DAPI (cyan), F-actin (blue), and mCherry-CD9 (red); scale bar = 10 μM). (**H**) Quantification of co-localization of nanoluciferase and mCherry-CD9. * indicates *p* < 0.05 (**I**) Nanoluciferase fused with Palmito-Tag 3 was similarly deposited within the extracellular vesicles. ALIX was used as a marker for extracellular vesicles (Original images can be found in Supplementary Materials). (**J**) Quantification of loading efficiency of NanoLuc with or without Palmito-Tag #3. (**K**,**M**) Quantification of the luciferase activity. A luciferase assay was performed to determine whether the addition of Palmito-Tag could compromise the endogenous function of the target protein. In cell lysate (**K**), extracellular vesicles (**L**), and recipient cells (**M**), luciferase activity was present. Compared to the control, nanoluciferase fused with the Palmito-Tag showed higher luciferase activity within the extracellular vesicle and recipient cells, consistent with its subcellular localization.

We then examined the possibility that the Palmito-Tag-containing EGFP could be carried over into the extracellular vesicles, which are known to originate from the multivesicular endosome or plasma membrane [20]. From the conditioned cell culture medium, we isolated extracellular vesicles and analyzed the presence of EGFP. Compared to the control, which were collected from HEK293T transfected with EGFP without any tag, those from the HEK293T expressing EGFP fused with various Palmito-Tags contained a relatively high level of EGFP, suggesting that the Palmito-Tag could direct EGFP to extracellular vesicles (Figure 3A,B), potentially by promoting their subcellular localization within the endosomes. Interestingly, there appears to be a strong positive correlation between the membrane association of EGFP and their deposition into the extracellular vesicles, as EGFP fused with either Palmito-Tags 3 or 7 was more likely to be found within the extracellular vesicles than those fused with Palmito-Tags 4 or 8 (Figure 3A,B). The presence of the Palmito-Tag not only enables EGFP to be loaded into the extracellular vesicles but also allows them to be transferred to another cell. We found that substantial portion of HUVEC cells treated with isolated extracellular vesicles from the HEK 293T cells expressing Palmito-tagged EGFP contains EGFP, suggesting that the Palmito-Tag could enable proteins to be transferred to other cells via extracellular vesicles (Figure 3C–E).

### 3.3. Palmito-Tag Alters Subcellular Localization Without Affecting the Biological Function of a Target Protein

To assess whether the Palmito-Tag could be used as a tool to direct subcellular localization of diverse proteins, we conjugated the Palmito-Tag 3 with nanoluciferase, as it was the most effective inducer of palmitoylation and altered the subcellular localization of EGFP. Upon addition of Palmito-Tag 3, we were able to detect nanoluciferase with Palmito-Tag, which was well co-localized with CD9-mCherry in the membranous structure (Figure 3F,G). Interestingly, nanoluciferase with Palmito-Tag appears to be incorporated into the extracellular vesicles (Figure 3I,J). Considering that untagged nanoluciferase is uniformly distributed within mammalian cells [21], it appears that the presence of Palmito-Tag may have altered their subcellular localization to the membranous structures within a cell.

The addition of the Palmito-Tag does not appear to interfere with the endogenous function of the target protein, as luminescence measurement using cell lysate shows nanoluciferase with the Palmito-Tag retains its enzymatic function. Similarly, we observed strong enzymatic activity within the extracellular vesicles harvested from the HEK293T cells expressing Palmito-Tag-containing nanoluciferase compared to those harvested from the control (Figure 3L). Consistent with this observation, Cre and β-galactosidase fused with Palmito-Tag showed similar alteration in their distribution pattern within the cells and could be loaded into extracellular vesicles (Figure S2B,C), hinting that proteins with Palmito-Tag could be loaded into the extracellular vesicles without perturbing their endogenous function.

Within the extracellular vesicle, it appears that nanoluciferase can be transferred to another cell while retaining its endogenous function. While HUVECs treated with extracellular vesicles containing media harvested from HEK293T transfected with the control construct did not exhibit any luminescence, those treated with extracellular vesicle-containing media harvested from HEK293T cells transfected with Palmito-Tag-fused nanoluciferase showed strong luminescence (Figure 3M). Therefore, it appears that Palmito-Tag could direct the deposition of a target protein to the extracellular vesicles, which would finally make it possible to deliver target proteins into other cells without affecting their endogenous function.

### 3.4. The Presence of Palmito-Tag Is Sufficient to Induce Ectopic Palmitoylation

**Figure 4:**
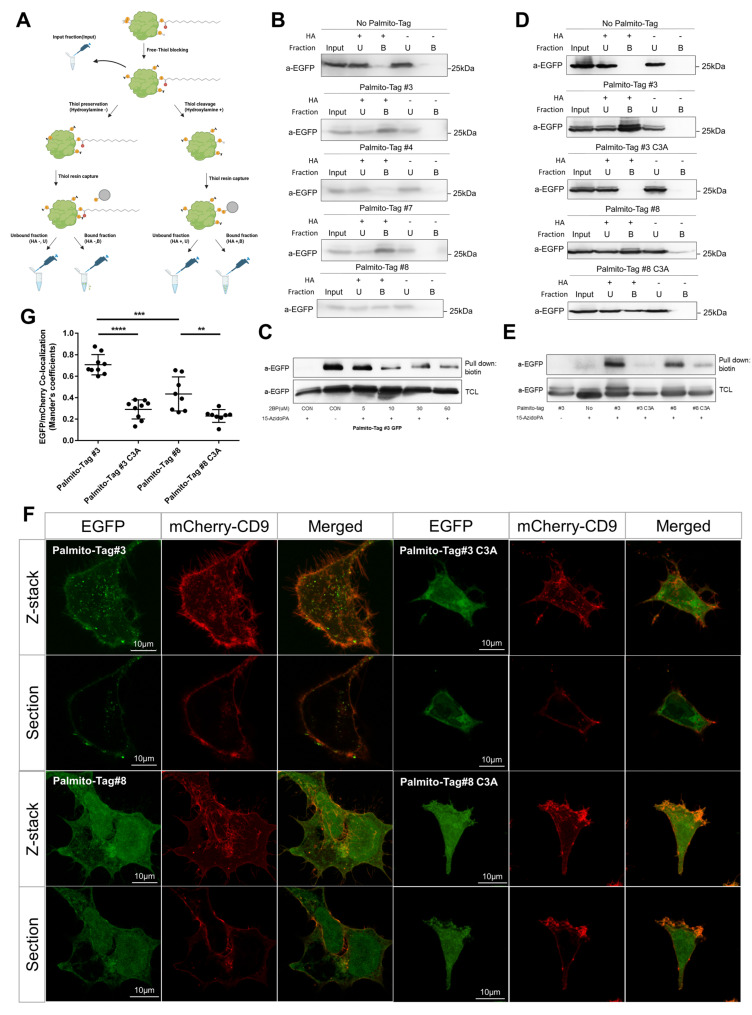
Palmito-Tag undergoes palmitoylation at the cysteine residue. (**A**) Schematic drawing of the acyl-biotin exchange assay. (**B**) To detect palmitoylation of EGFP with Palmito-Tag, an acyl-biotin exchange assay was performed. EGFP fused with different Palmito-Tags showed varying degrees of palmitoylation. (**C**) Inhibition of palmitoylation using 2-bromopalmitate abrogates palmitoylation within the Palmito-Tag. (**D**,**G**) To identify palmitoylation of Palmito-Tag at the Cys residue, the third position of Cys was substituted as Ala (C3A). The C3A mutant of Palmito-Tag shows a significantly reduced level of palmitoylation in both the acyl-biotin exchange assay (**D**) and click chemistry (**E**) (Original images can be found in Supplementary Materials). (**F**) Palmito-Tag with C3A substitution shows a significantly decreased in its ability to localize protein on membranous structures like the plasma membrane and endosomes. (G) Quantification of co-localization of EGFP and mCherry-CD9. ** indicates *p* < 0.01, *** indicates *p* < 0.001, **** indicates *p* < 0.0001.

To validate that the Palmito-Tags are indeed used as a substrate for palmitoylation, we examined the level of S-palmitoylation in EGFP fused with the Palmito-Tag using an acyl-biotin exchange assay and click chemistry (Figure 4A). We found that EGFPs with Palmito-Tags 3 or 7, which preferentially localized to the plasma membrane, were heavily palmitoylated (Figure 4B), similar to GAP43-tagged EGFP, which is known to undergo palmitoylation (Figure S2A). In comparison, EGFP fused with the Palmito-Tags 4 or 8, which showed partial localization to the plasma membrane, exhibited a much lower level of palmitoylation (Figure 4B), hinting at a correlation between the level of palmitoylation and the membrane localization of a target protein. In addition, nanoluciferase, Cre, and β-galactosidase fused with Palmito-Tag 3 appeared to undergo palmitoylation (Figure S2D). Treatment with 2-bromopalmitate (2-BP) abrogated palmitoylation of the target proteins induced by Palmito-Tag, further supporting the notion that Palmito-Tag could be used as a substrate for palmitoylation (Figure 4C). To assess whether the palmitoylation of EGFP is dependent on the Cys residues within Palmito-Tag, we performed site-directed mutagenesis to alter the sequence of Palmito-Tags 3 and 8 and compared the levels of palmitoylation (Figure 4D,E). Substitution of the Cys residue at the 3rd position of the Palmito-Tag 3 to Ala (Palmito-Tag 3 C3A) abrogated the palmitoylation of the EGFP with those tags and diminished the localization of EGFP into membranous structure (Figure 4F), indicating that Cys at the third position is the target residue for the palmitoylation.

## 4. Discussion

In this report, we show that the Palmito-Tag could direct subcellular localization of the target proteins in an S-palmitoylation-dependent manner. By undergoing palmitoylation at its cysteine residue, Palmito-Tag can induce palmitoylation of the target proteins without disrupting the endogenous function of the target proteins. Considering that there is an unmet need for a synthetic tag that could emulate the dynamic trafficking of endogenous proteins, we believe that the Palmito-tag could be used as a synthetic substrate for palmitoylation and direct the tagged protein into the membranous structures within the cell. The Palmito-Tags we designed contain both Cys residues, which could serve as a substrate for palmitoylation, and Gly residues, which could undergo myristoylation. While acyl biotin exchange or click chemistry-based essays [22] could support the idea that Palmito-Tag induces ectopic palmitoylation within the target proteins, we cannot formally exclude the possibility that the myristoylation of Gly residues also contributes to the alteration of the subcellular localization of the target proteins, as myristoylation precedes palmitoylation in certain proteins. To further support the palmitoylation-dependent effects of our synthetic tag, we utilized site-directed mutagenesis and further validated the essential contribution of Cys residues within the Palmito-Tag. However, more precise analyses, such as a specialized mass spectrometry-based detection [23], should be warranted to assess the potential contribution of myristoylation in the Gly residues.

Although we could not determine the percentages of proteins with Palmito-Tag moving into EVs to the percentage of endosomes or membranes, Palmito-Tag efficiently enriched the deposition of the target protein in the endosomes compared to the control. Consequently, it could increase the loading of the target protein into the extracellular vesicles, which are thought to originate from the endosome [20]. As the efficacy of loading proteins of interest into the extracellular vesicles is the key rate-limiting step for developing the extracellular vesicle-based drug delivery carriers [24], our new synthetic polypeptide protein tag could help us facilitate the application of the extracellular vesicles for therapeutic purposes. To date, the consensus sequence for S-palmitoylation has not been fully identified, despite its biological importance [25]. However, a number of bioinformatics tools have been developed to deduce the consensus sequence for S-palmitoylation and reliably predict this elusive post-translational modification [26,27]. We find that our synthetic sequence is distinct from the previously reported DHHC sequences, which serve as targets for DHHC13 and DHHC17, arguably the most well-characterized target sequence for auto-palmitoylation [12]. By using a brute-force approach to analyze the sequences of known palmitoylated proteins around the palmitoylated Cys residue, we experimentally determined a potential consensus sequence for S-palmitoylation spanning 11 amino acids. Therefore, the sequence of the Palmito-Tag differs from the previously identified internal target sequence for auto-palmitoylation in DHHC proteins. While we cannot determine whether sequence differences between the Palmito-Tag and previously reported recognition motifs for auto-palmitoylation in DHHC13 and DHHC17 would reflect the innate molecular mechanisms whereby palmitoylation occurs, it is certain that the novel synthetic sequence we developed could effectively induce palmitoylation. Further analyses are warranted to precisely determine the difference between recognition motifs for auto-palmitoylation and exogenous palmitoylation. As we chose the sequence for the Palmito-Tag based on an unbiased brute force approach, we speculate that our novel synthetic sequence might resemble a previously unidentified amino acid sequence that could serve as a target for palmitoylation. Our results suggest that the presence of a positively charged amino acid in the seventh position is essential to ensuring effective palmitoylation (Palmito-Tags 3, 7 versus 4, 8), while the amino acid charge of the ninth position has minimal effects on palmitoylation of Palmito-Tag (i.e., Palmito-Tag 1 versus Palmito-Tag 3). Therefore, it appears that our analyses extend the consensus sequence for S-palmitoylation and reveal the previously underappreciated roles of the charged amino acid within the milieu.

Since palmitoylation is a reversible modification that could facilitate dynamic trafficking of the target proteins [6], we speculate that the Palmito-Tag exerts similar effects on the target proteins. Not only could the Palmito-Tag effectively induce palmitoylation of the target proteins and therefore alter their subcellular localization, but it could also direct the deposition of the target proteins into the extracellular vesicles. In addition, due to the small size of the Palmito-Tag, its addition to the N-terminus of the target protein does not appear to interfere with the endogenous function of the target protein. We find that the addition of the Palmito-Tag to a cytoplasmic enzyme did not compromise the endogenous activity, rendering it an effective tag to direct subcellular localization of a target protein. Combined, these two features of Palmito-Tag raise the possibility that it could be applied to deliver a target protein to achieve specific therapeutic effects, and extracellular vesicles could be used as a drug delivery system to introduce therapeutic molecules into the recipient cells [28,29]. We found drastic changes in subcellular localization of proteins with the Palmito-Tag into extracellular vesicles and membranous structures in HEK293T, utilization of this synthetic tag in other model organisms such as yeast should be further analyzed considering different regulatory enzymes for palmitoylation [30,31]. Since we utilized limited methods such as 2-BP and site-directed mutagenesis to regulate palmitoylation of the Palmito-Tag, further investigation on the detailed mechanisms of palmitoylation and depalmitoylation dynamics of the Palmito-Tag-conjugated proteins is warranted prior to such consideration.

## 5. Conclusions

De novo synthetic tags, which we named Palmito-Tag, allowed the modulation of subcellular localization for protein without affecting its innate function through S-palmitoylation. Tagged proteins could be more efficiently loaded into extracellular vesicles and transferred to another cell. This new short synthetic tag would be used for translocation of protein into membranous structure with minimal effects on protein function.

## 6. Patents

Haekwan Jeong. Korean Intellectual Property Office (KIPO). KR, Patent 10-2023-0055620, 27 April 2023.

## Data Availability

Data are contained within the article or Supplementary Material.

## Supplementary Materials

The following supporting information can be downloaded at: https://www.mdpi.com/article/10.3390/biom15081076/s1, Figure S1: a subset of Palmito-Tags can induce membrane association of the target protein; Figure S2: target proteins fused with the Palmito-Tag undergoes palmitoylation and can be loaded into the extracellular vesicles; Table S1: sequences of primers used for cloning is; Table S2: analysis results of predicted palmitoylated-sites and consensus amino acid sequence of proteins loaded in L-EVs and S-EVs.

## Abbreviations

CD9	Clusters of differentiation 9
EGFP	Enhanced green fluorescent protein
HEK293T	Human embryonic kidney 293T
HUVEC	Human umbilical vein endothelial cell
GAP43	Growth associated protein 43
PAT/DHHC	Palmitoyl-acyltransferases
PPT	Palmitoyl protein thioesterases
