# Supplementary material for: A Novel Synthetic Tag Induces Palmitoylation and Directs the Subcellular Localization of Target Proteins
Source: Biomolecules. 2025 Jul 25;15(8):1076. doi: 10.3390/biom15081076 (PMC12383751; doi:10.3390/biom15081076)
Supplement: Supplementary file 1 [file biomolecules-15-01076-s001.zip › Supplementary figure edited.docx]

**SUPPLEMENTARY MATERIALS**

**SUPPLEMENTARY TABLES**

**Table S1**

Sequences of primers used for cloning is listed.

**Table S2**

Analysis results of predicted palmitoylated-sites and consensus amino acid sequence of proteins loaded in L-EVs and S-EVs. Each column in the table implies as follows. Palm-predicted site: Region of proteins where palmitoylation is induced; N (N-terminus), M (Middle part of the protein), C (C-terminus). No. predicted site (GPS-palm): Total number of palmitoylated sites in a protein according to the score analyzed using GPS-Palm program. The first number in a column means highly predicted palmitoylated-sites; Score>0.9. Second number means more comprehensively expected sites; Score>0.75. 25 amino acids sequence in N-terminus of proteins loaded in L-EVs and S-EVs are notated. Amino acids sequence in C-terminus also notated when predicted palmitoylated-sites are in C-terminus of protein. Colored cells mean as follows. Yellow: Predicted palmitoylated-cysteine. Green: Predicted myristoylated-glycine. Blue: Positively charged amino acids. Red: Negatively charged amino acids.


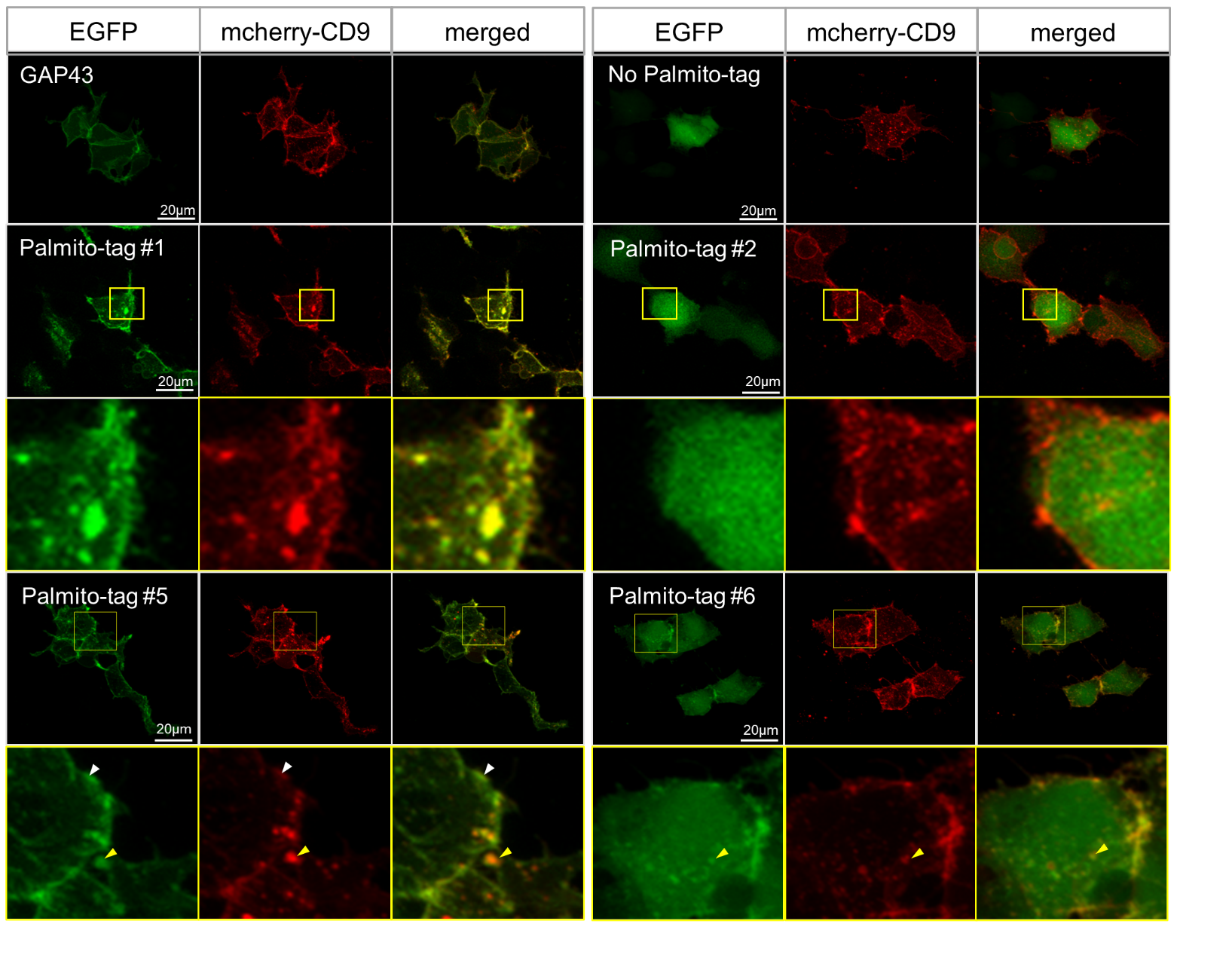
**SUPPLEMENTARY FIGURES**

**Figure S1: A subset of Palmito-Tags can induce membrane association of the target protein**

Subcellular localization of the EGFP tagged with different Palmito-Tags shown with GAP43 tagged EGFP as a positive control and non-tagged EGFP as a negative control. EGFP with Palmito-Tag shows increased co-localization with mCherry-CD9 in various levels depends on the tag while EGFP without tag shows no overlapping localization with mCherry-CD9. Areas within the yellow square were shown in higher magnification. (Colors: EGFP-Palmito-Tag (green) and mCherry-CD9 (green); Scale bar=20μM).


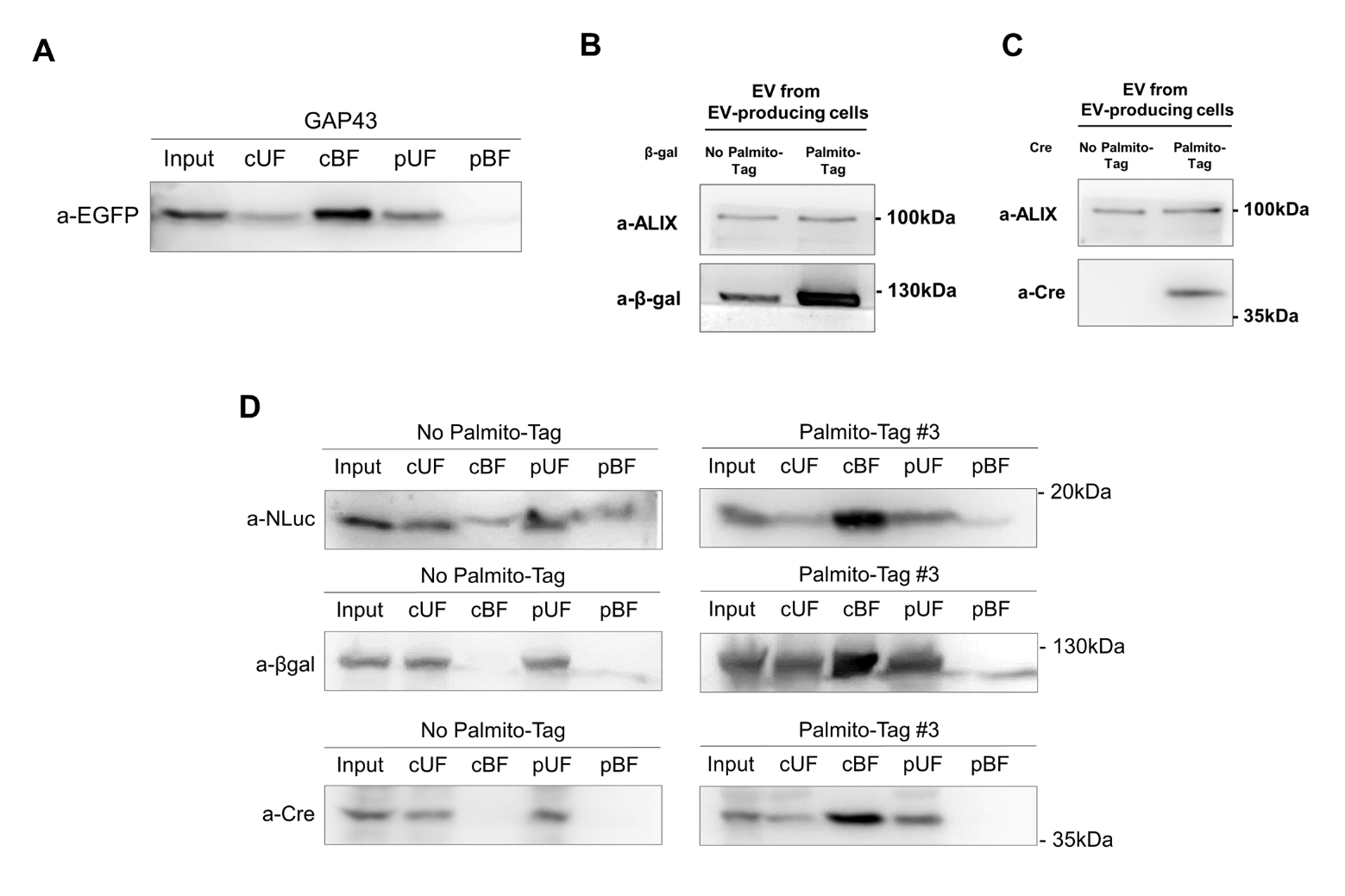


**Figure S2: Target proteins fused with the Palmito-Tag undergoes palmitoylation and can be loaded into the extracellular vesicles**

(**A**) EGFP with GAP43 sequences undergoes palmitoylation

(**B**-**C**) Extracellular vesicles were purified from the conditioned media of the Palmito-Tag-fused β-gal or cre expressing HEK293T cells. Target proteins with Palmito-Tag effectively loaded into extracellular vesicles. ALIX was used as markers for extracellular vesicles.

(**D**) Acyl-biotin exchange assay was performed to identify palmitoylation of various target proteins with or without Palmito-Tag. Palmito-Tag effectively induces palmitoylation of target proteins like nanoluciferase, β-gal and Cre.
