# Supplementary material for: A Novel Synthetic Tag Induces Palmitoylation and Directs the Subcellular Localization of Target Proteins
Source: Biomolecules. 2025 Jul 25;15(8):1076. doi: 10.3390/biom15081076 (PMC12383751; doi:10.3390/biom15081076)

Original images for western blot

Figure.3A [1]

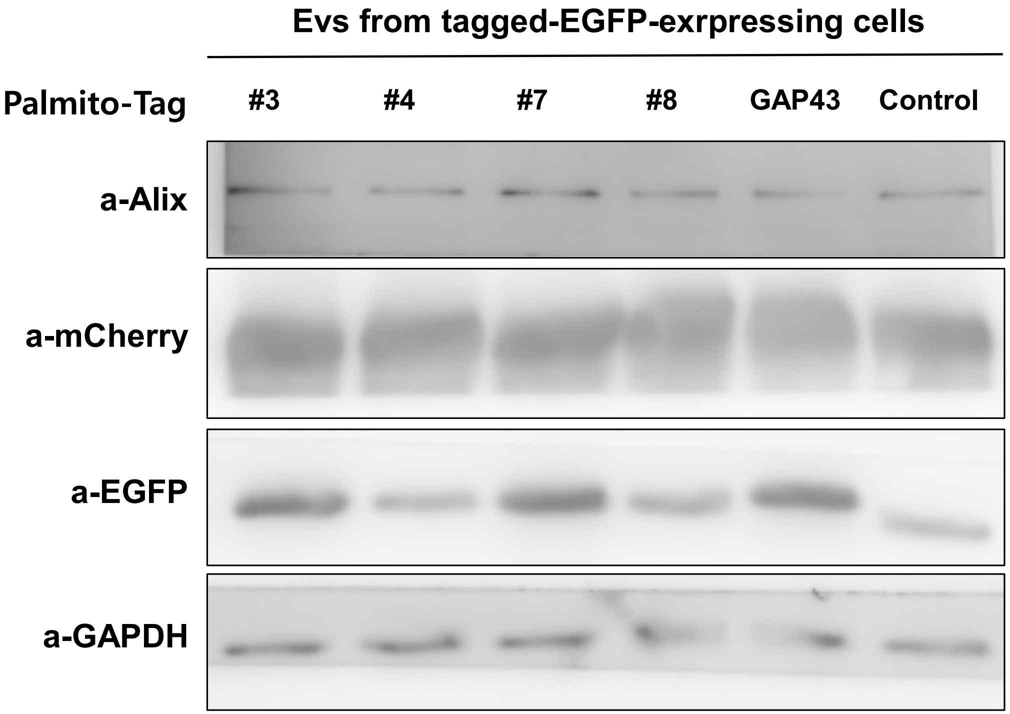
Evs from tagged-EGFP-exrpressing cells
Palmito-Tag
#3
#4
#7
#8
GAP43
Control
a-Alix
a-mCherry
a-EGFP
a-GAPDH

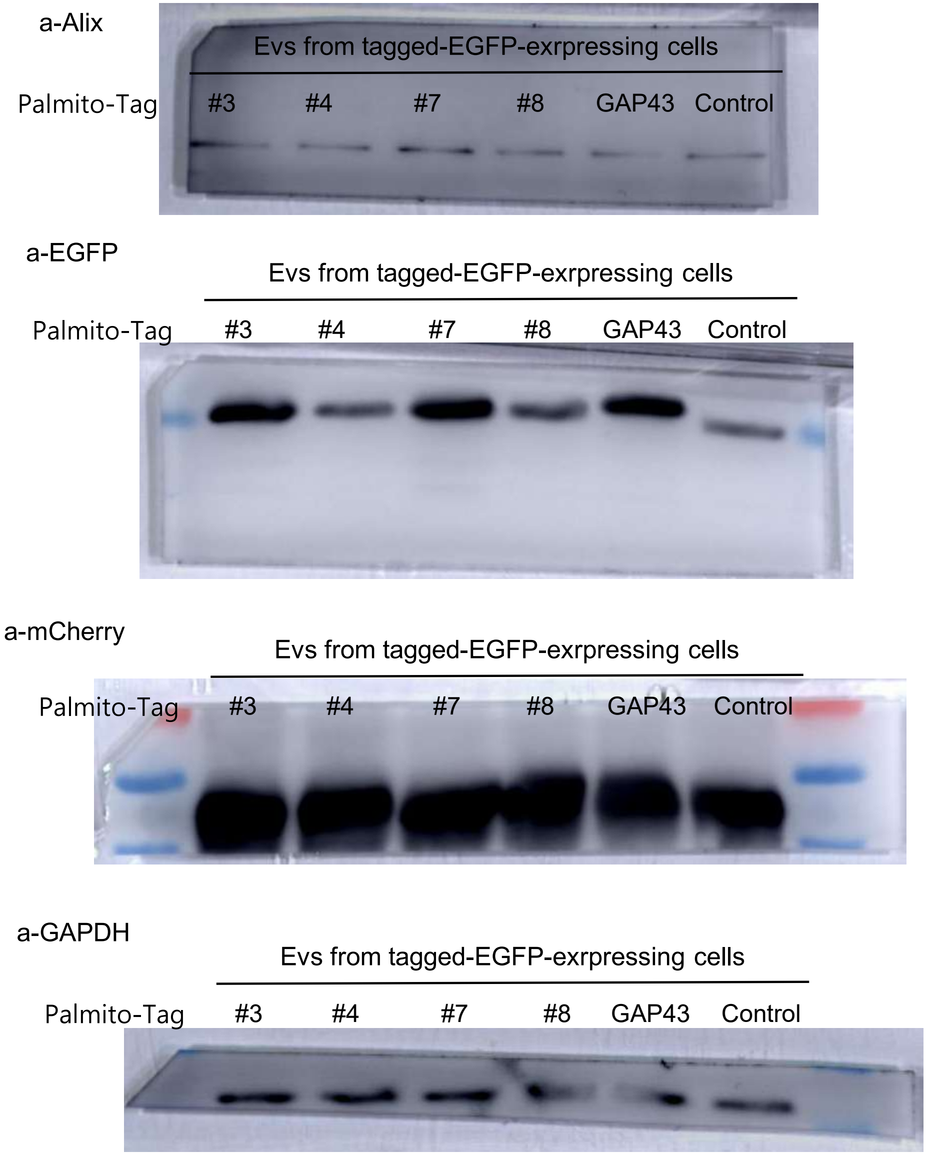
a-Alix
Evs from tagged-EGFP-exrpressing cells
Palmito-Tag
#3
#4
#7
#8
GAP43
Control
a-EGFP
Evs from tagged-EGFP-exrpressing cells
Palmito-Tag
#3
#4
#7
#8
GAP43
Control
a-mCherry
Evs from tagged-EGFP-exrpressing cells
Palmito-Tag
#3
#4
#7
#8
GAP43
Control
a-GAPDH
Evs from tagged-EGFP-exrpressing cells
Palmito-Tag
#3
#4
#7
#8
GAP43
Control

Figure.3A [2]

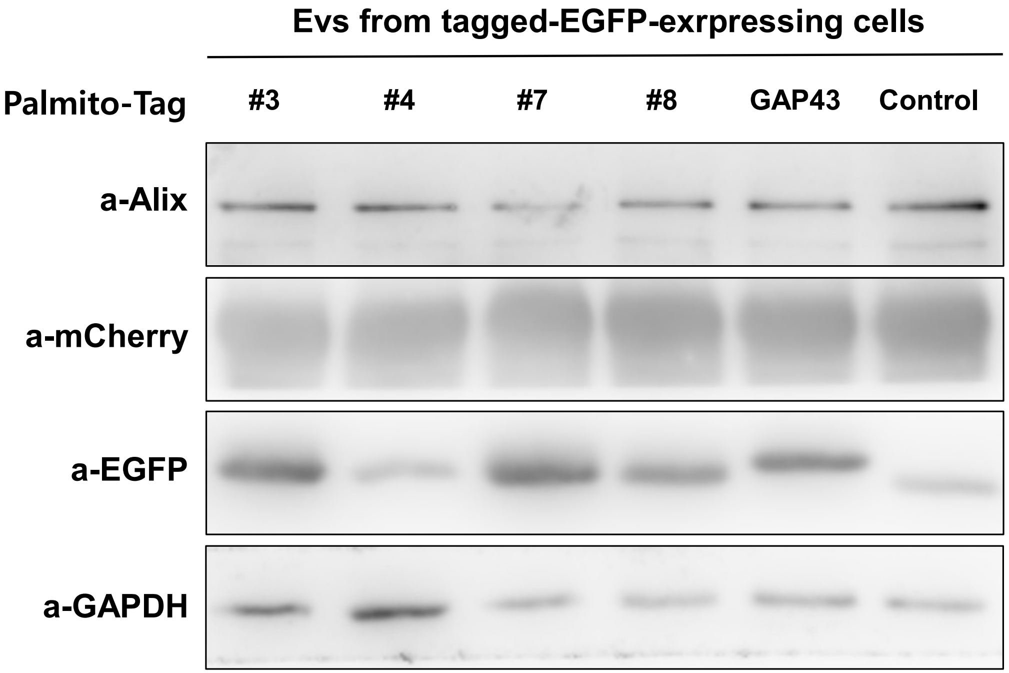
Evs from tagged-EGFP-exrpressing cells
Palmito-Tag
#3
#4
#7
#8
GAP43
Control
a-Alix
a-mCherry
a-EGFP
a-GAPDH

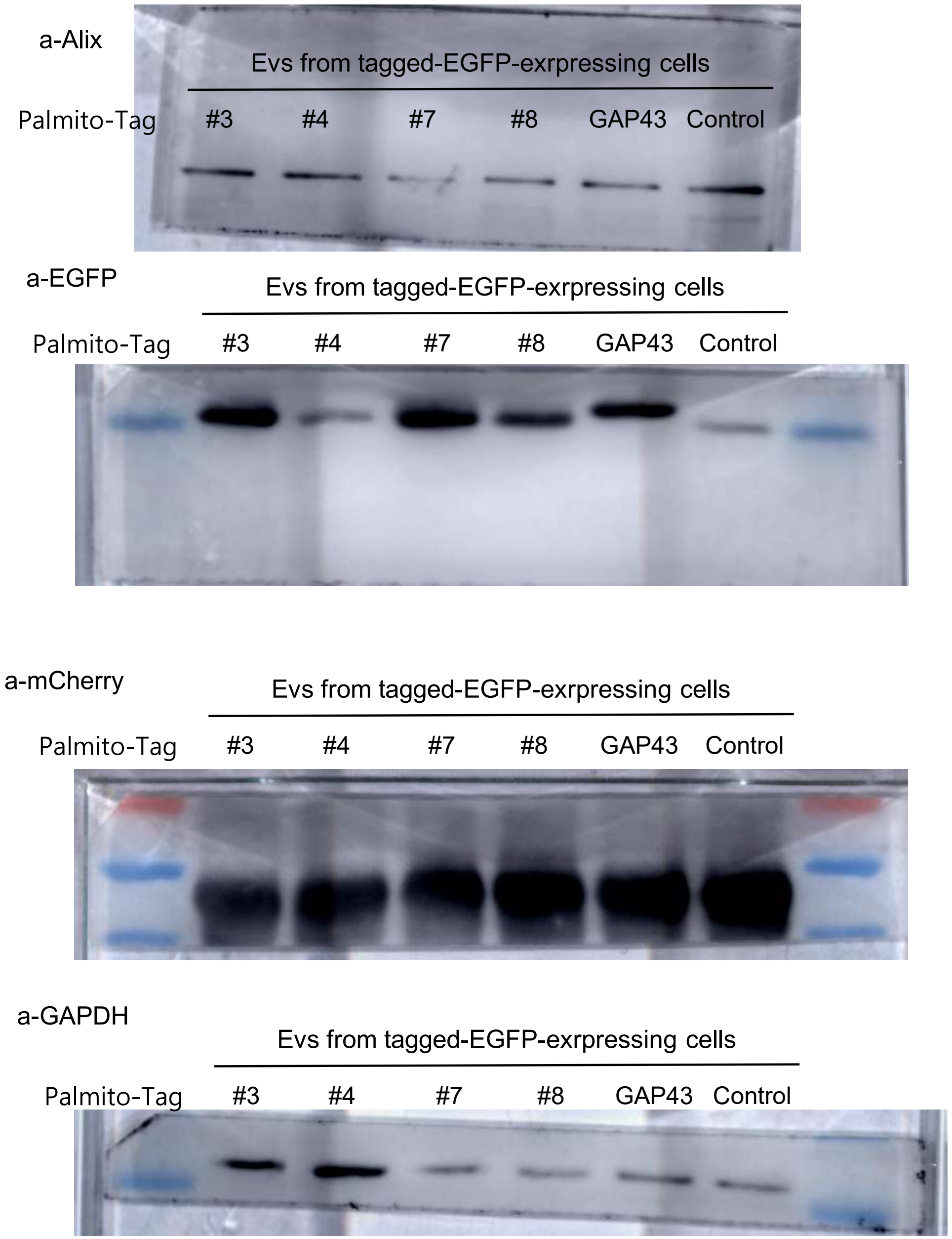
a-Alix
Evs from tagged-EGFP-exrpressing cells
Palmito-Tag
#3
#4
#7
#8
GAP43
Control
a-EGFP
Evs from tagged-EGFP-exrpressing cells
Palmito-Tag
#3
#4
#7
#8
GAP43
Control
a-mCherry
Evs from tagged-EGFP-exrpressing cells
Palmito-Tag
#3
#4
#7
#8
GAP43
Control
a-GAPDH
Evs from tagged-EGFP-exrpressing cells
Palmito-Tag
#3
#4
#7
#8
GAP43
Control

Figure.3A [3]

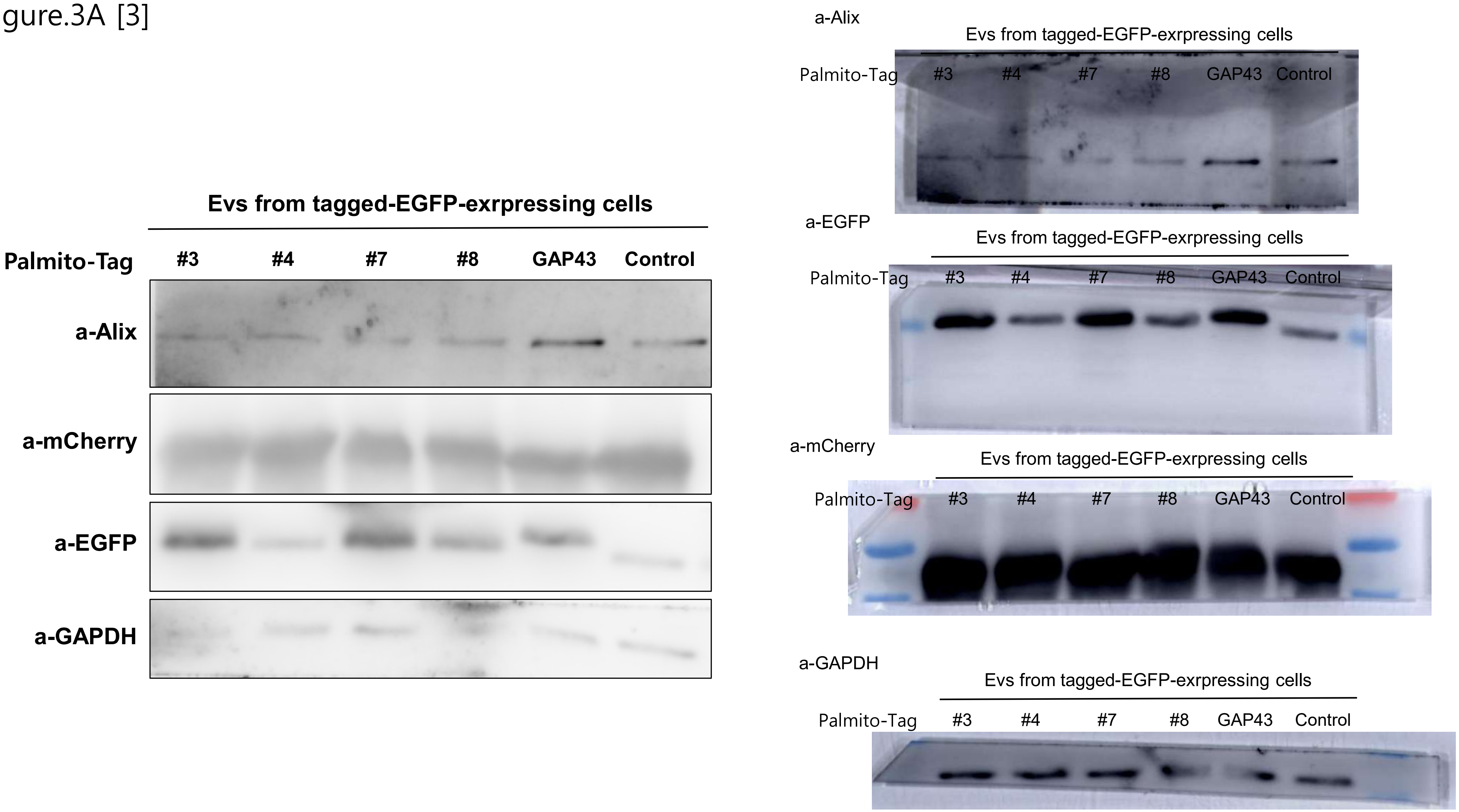
Evs from tagged-EGFP-exrpressing cells
Palmito-Tag
#3
#4
#7
#8
GAP43
Control
a-Alix
a-mCherry
a-EGFP
a-GAPDH
a-Alix
Evs from tagged-EGFP-exrpressing cells
Palmito-Tag #3 #4 #7 #8 GAP43 Control
a-EGFP
Evs from tagged-EGFP-exrpressing cells
Palmito-Tag #3 #4 #7 #8 GAP43 Control
a-mCherry
Evs from tagged-EGFP-exrpressing cells
Palmito-Tag #3 #4 #7 #8 GAP43 Control
a-GAPDH
Evs from tagged-EGFP-exrpressing cells
Palmito-Tag #3 #4 #7 #8 GAP43 Control

Figure.3A [4]

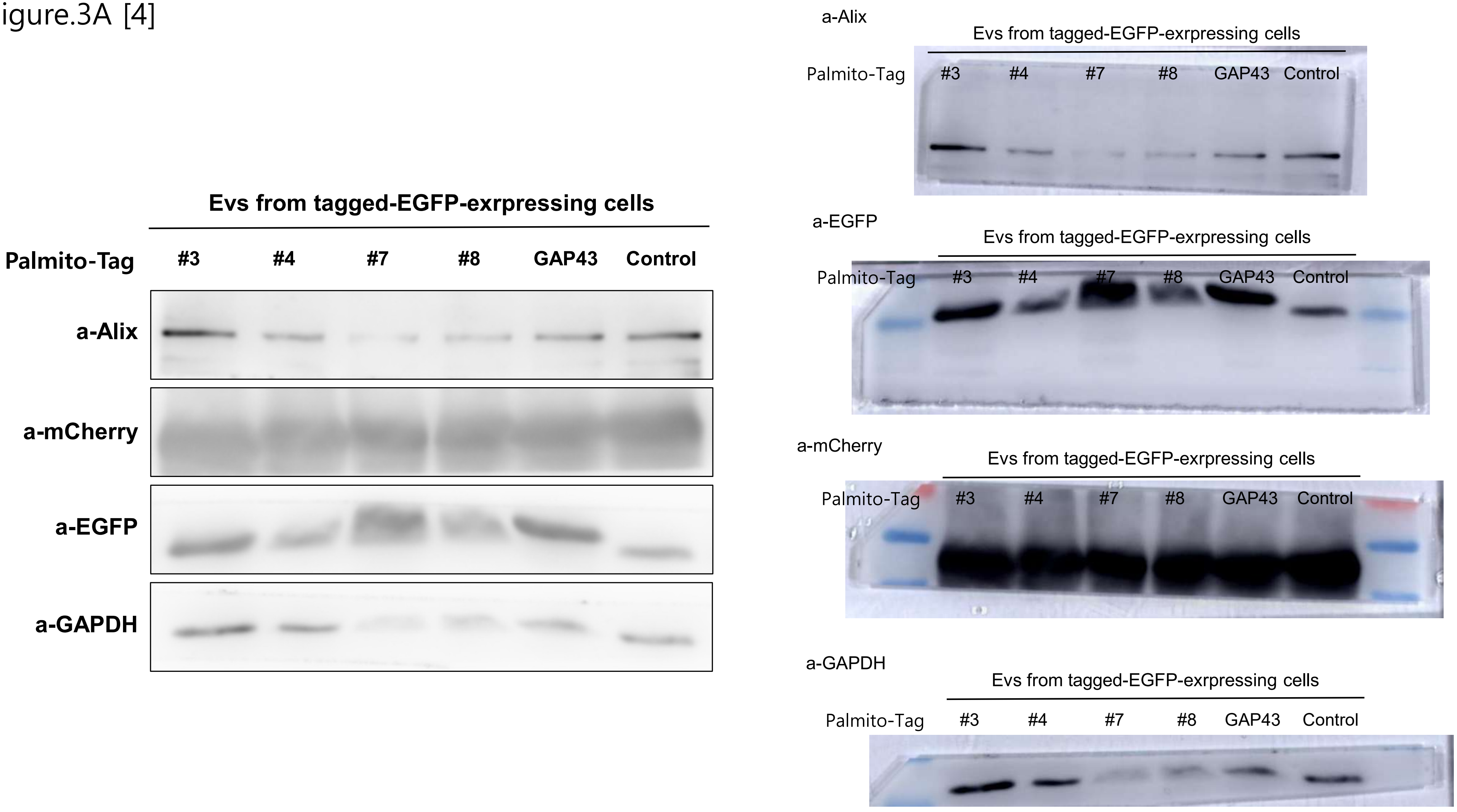
Evs from tagged-EGFP-exrpressing cells
Palmito-Tag
#3
#4
#7
#8
GAP43
Control
a-Alix
a-mCherry
a-EGFP
a-GAPDH
a-Alix
Evs from tagged-EGFP-exrpressing cells
Palmito-Tag
#3
#4
#7
#8
GAP43
Control
a-EGFP
Evs from tagged-EGFP-exrpressing cells
Palmito-Tag
#3
#4
#7
#8
GAP43
Control
a-mCherry
Evs from tagged-EGFP-exrpressing cells
Palmito-Tag
#3
#4
#7
#8
GAP43
Control
a-GAPDH
Evs from tagged-EGFP-exrpressing cells
Palmito-Tag
#3
#4
#7
#8
GAP43
Control

Figure.3I [1]

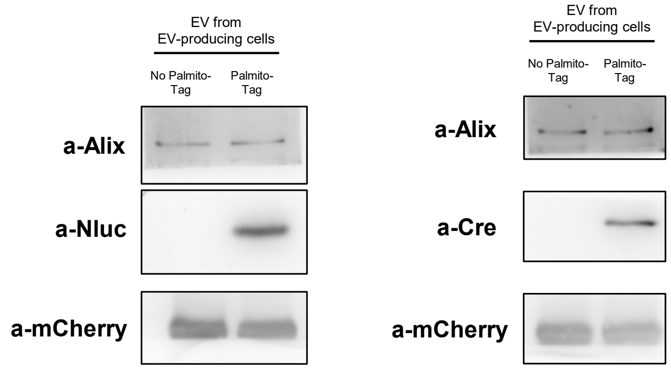

a-Nluc

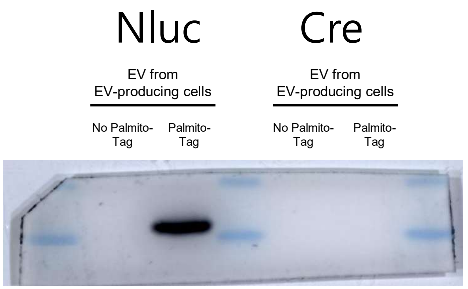

a-mCherry

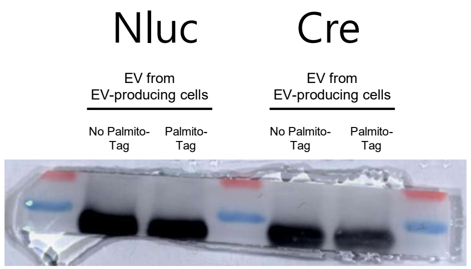

a-Cre

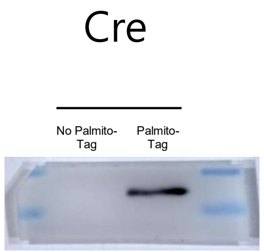

Figure.3I [2]

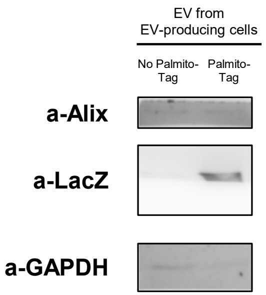

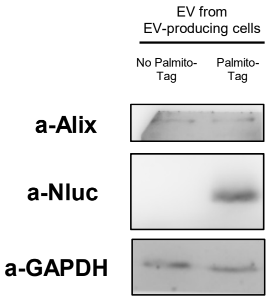

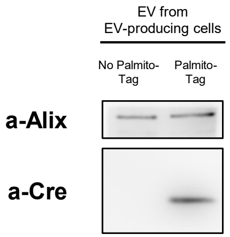

a-LacZ

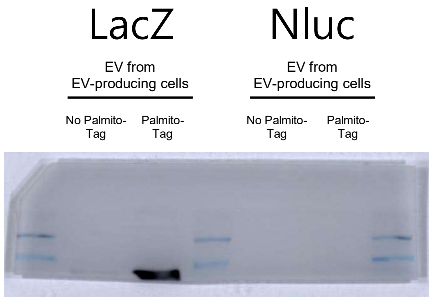

a-Nluc

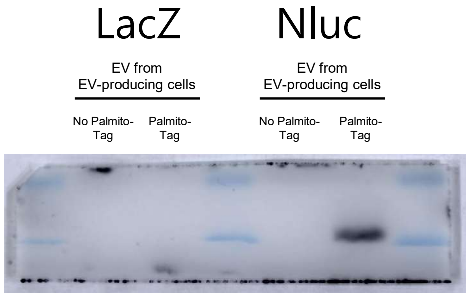

a-GAPDH

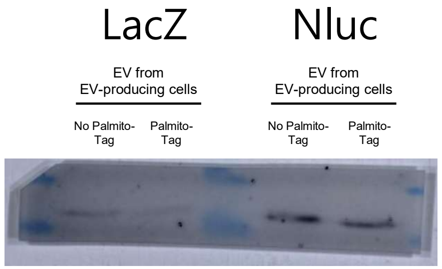

a-Cre

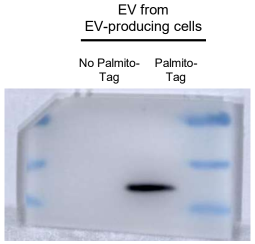

Figure.3I [3]

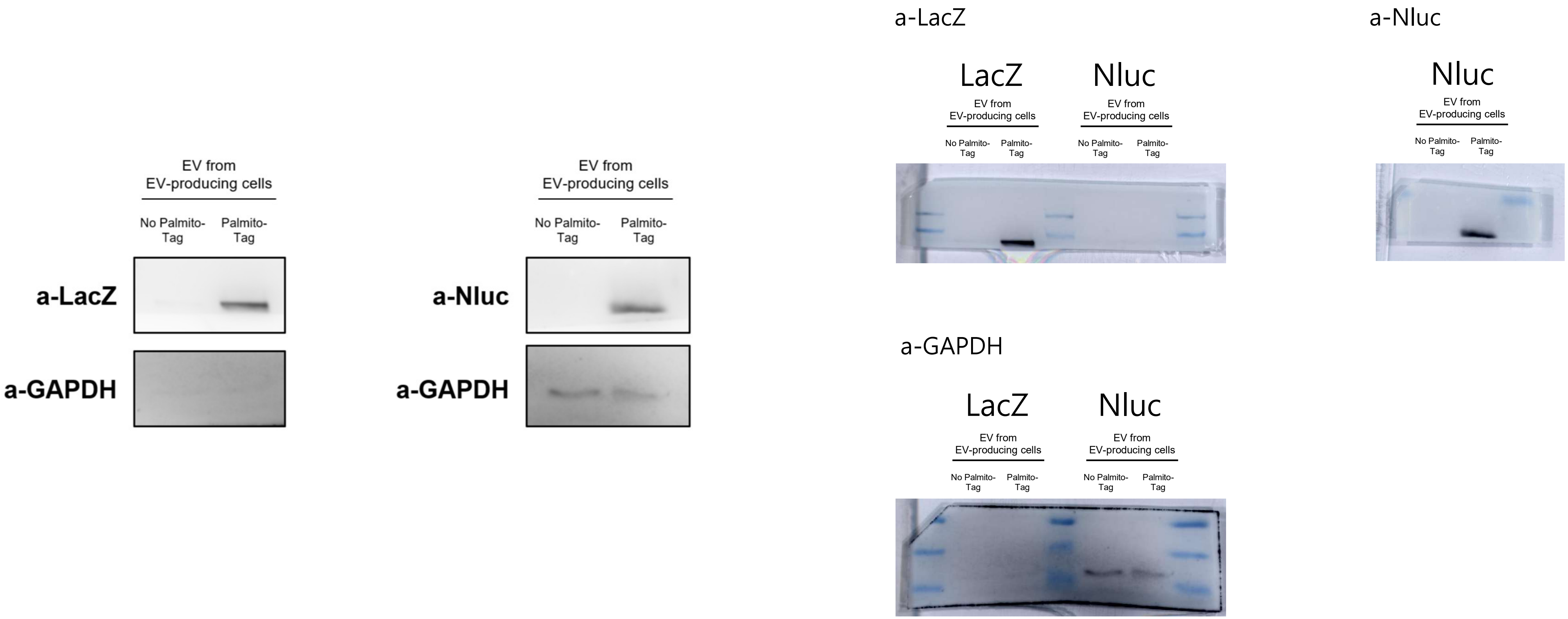

EV from
EV-producing cells
No Palmito-
Tag
Palmito-
Tag
a-LacZ
a-GAPDH
EV from
EV-producing cells
No Palmito-
Tag
Palmito-
Tag
a-Nluc
a-GAPDH
a-LacZ
LacZ
EV from
EV-producing cells
No Palmito-
Tag
Palmito-
Tag
Nluc
EV from
EV-producing cells
No Palmito-
Tag
Palmito-
Tag
a-Nluc
Nluc
EV from
EV-producing cells
No Palmito-
Tag
Palmito-
Tag
a-GAPDH
LacZ
EV from
EV-producing cells
No Palmito-
Tag
Palmito-
Tag
Nluc
EV from
EV-producing cells
No Palmito-
Tag
Palmito-
Tag

Figure.4B

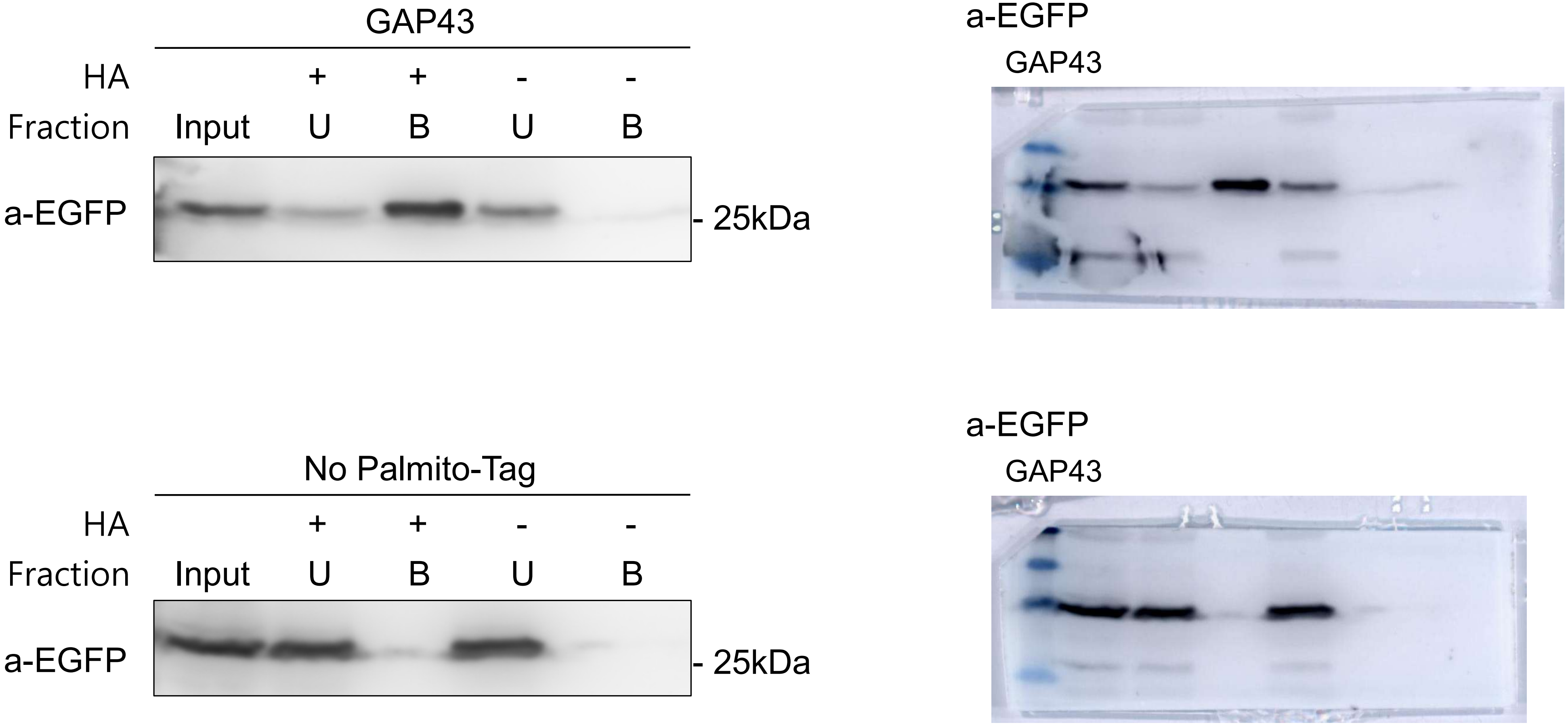

GAP43
HA + + - -
Fraction Input U B U B
a-EGFP
- 25kDa
a-EGFP
GAP43
No Palmito-Tag
HA + + - -
Fraction Input U B U B
a-EGFP
- 25kDa
a-EGFP
GAP43

Figure.4B

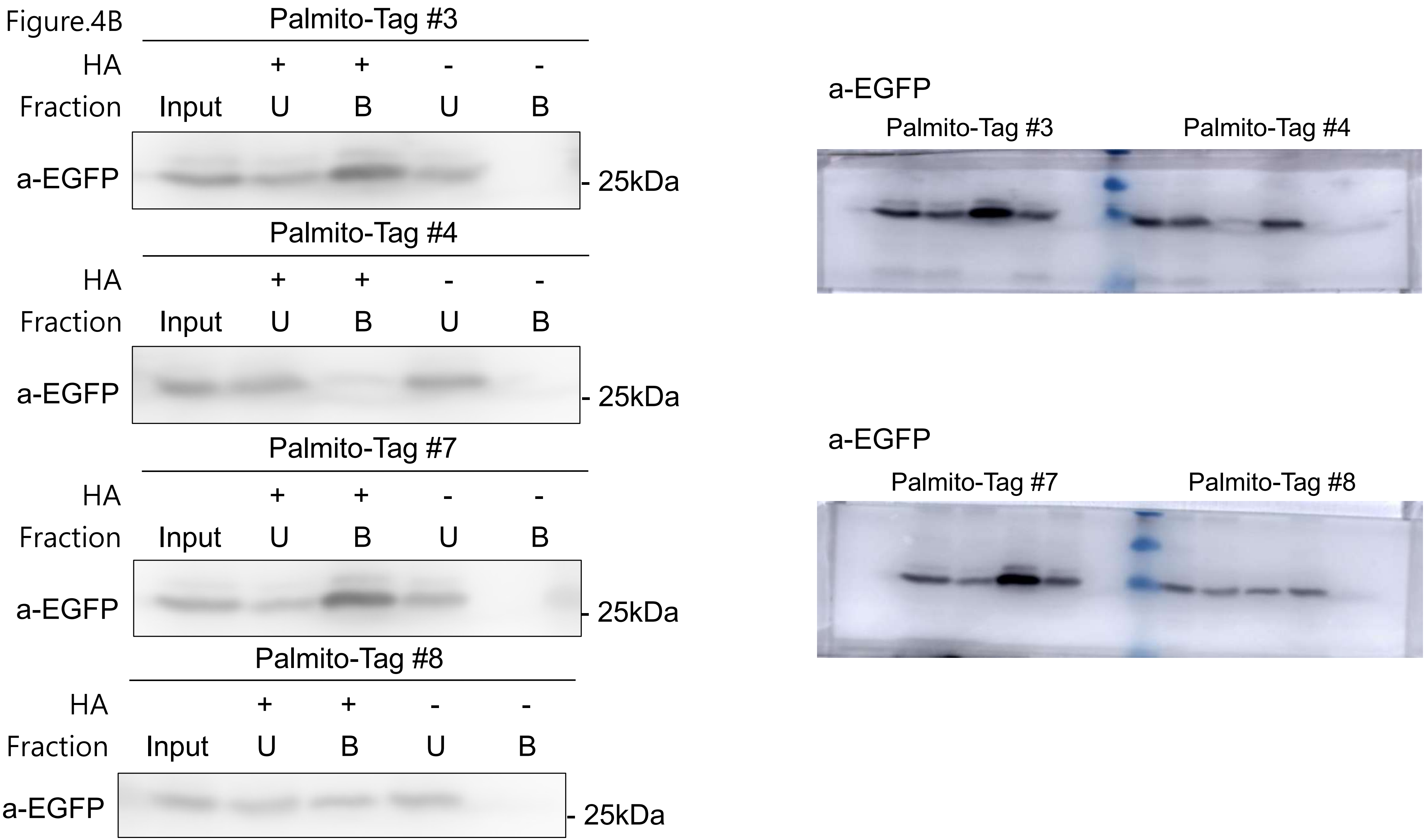
Palmito-Tag #3
HA + + - -
Fraction Input U B U B
a-EGFP
25kDa
Palmito-Tag #4
HA + + - -
Fraction Input U B U B
a-EGFP
25kDa
Palmito-Tag #7
HA + + - -
Fraction Input U B U B
a-EGFP
25kDa
Palmito-Tag #8
HA + + - -
Fraction Input U B U B
a-EGFP
25kDa
a-EGFP
Palmito-Tag #3
Palmito-Tag #4
a-EGFP
Palmito-Tag #7
Palmito-Tag #8

Figure.4C [1]

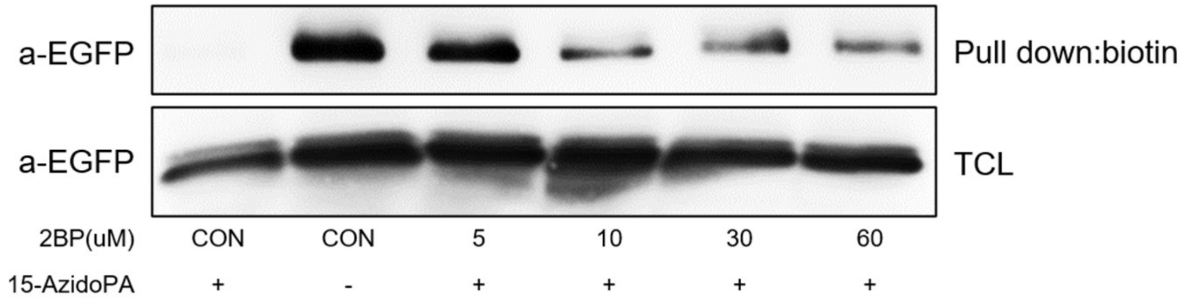

Pull down-EGFP(25kDa)

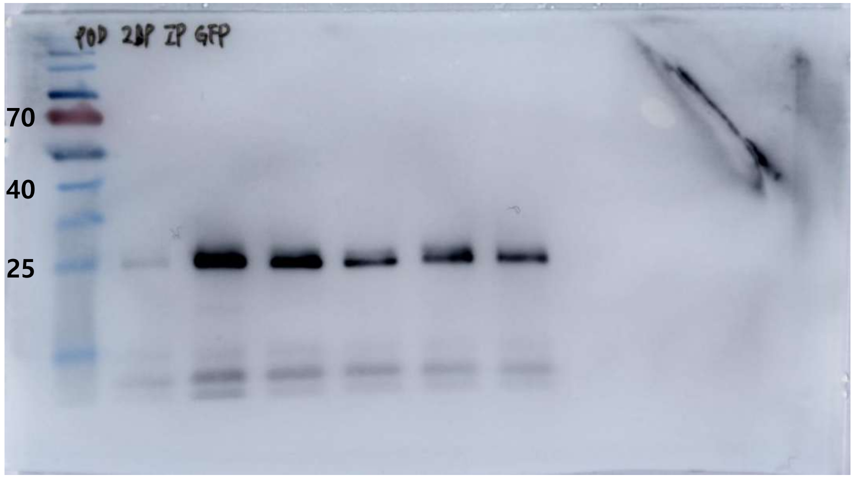

TCL-EGFP(25kDa)

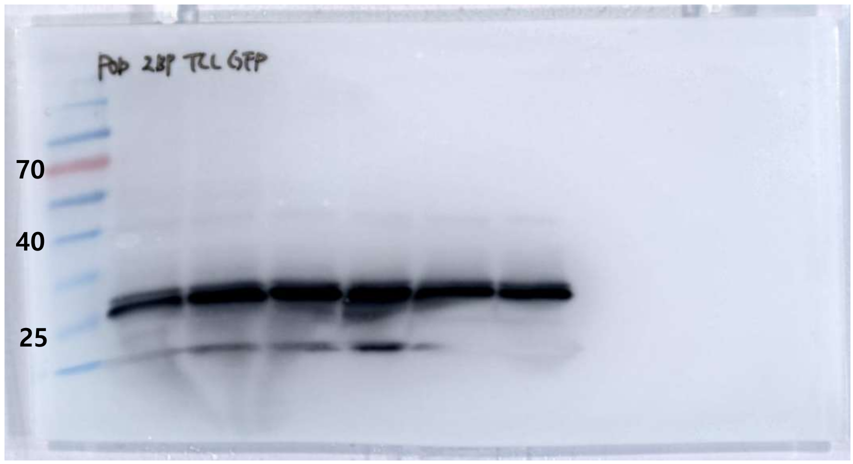

Figure.4C [2]

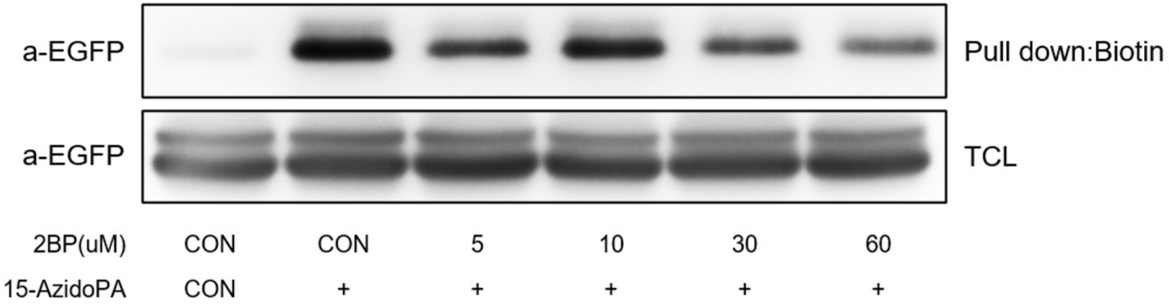

Pull down-EGFP(25kDa)

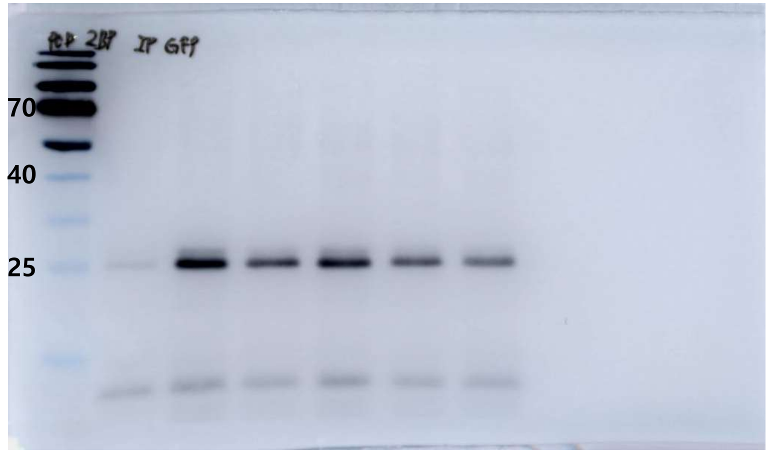

TCL-EGFP(25kDa)

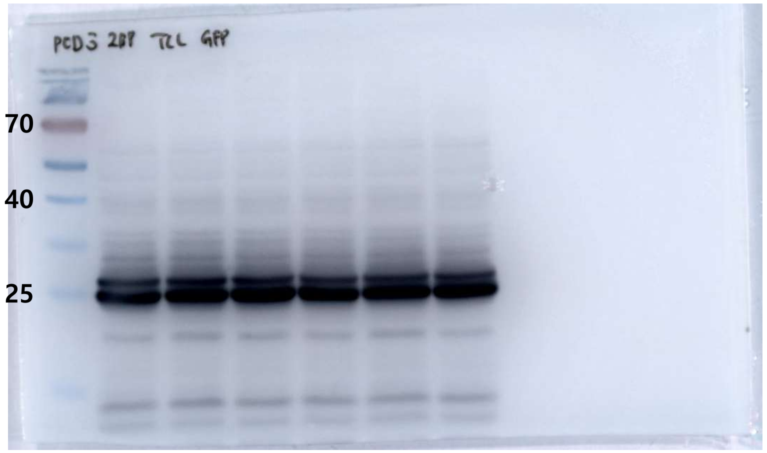

Figure.4C [3]

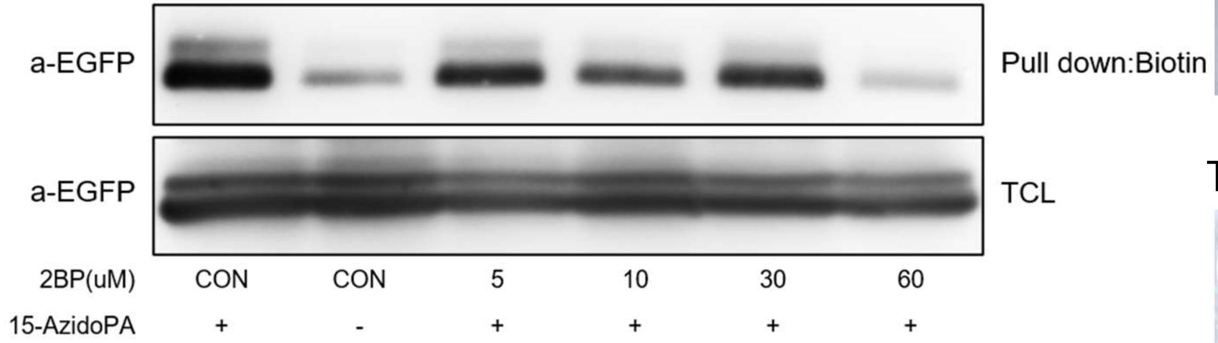

Pull down-EGFP(25kDa)

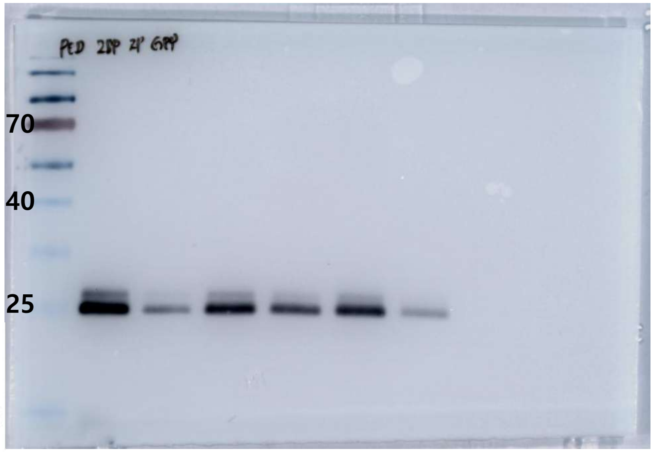

TCL-EGFP(25kDa)

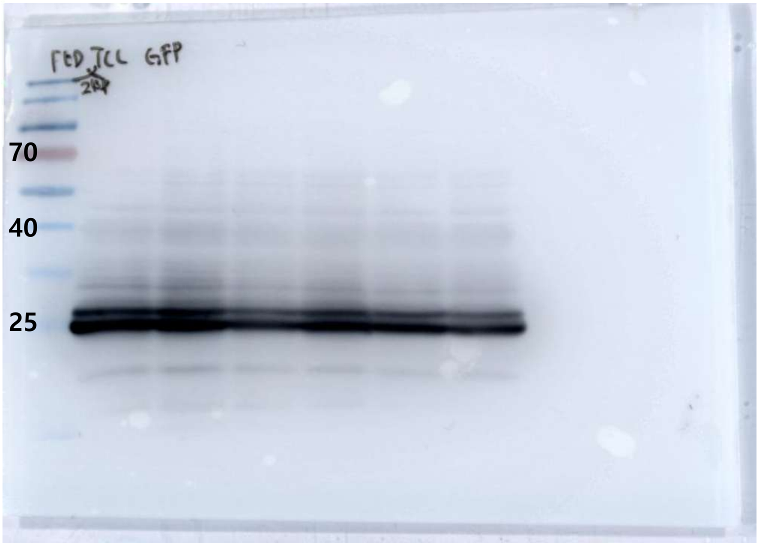

Figure.4D [1]

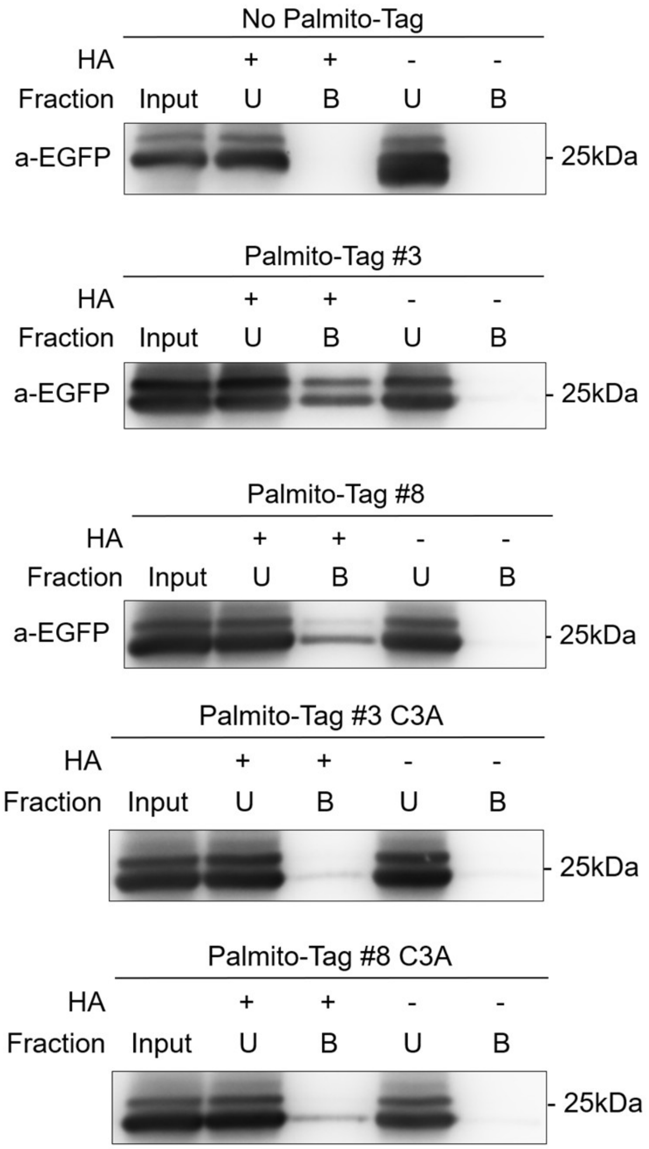

NO-Palmito-Tag a-EGFP

Palmito-Tag #3 a-EGFP

Palmito-Tag #8 a-EGFP

Palmito-Tag #3 C3A a-EGFP

Palmito-Tag #8 C3A a-EGFP

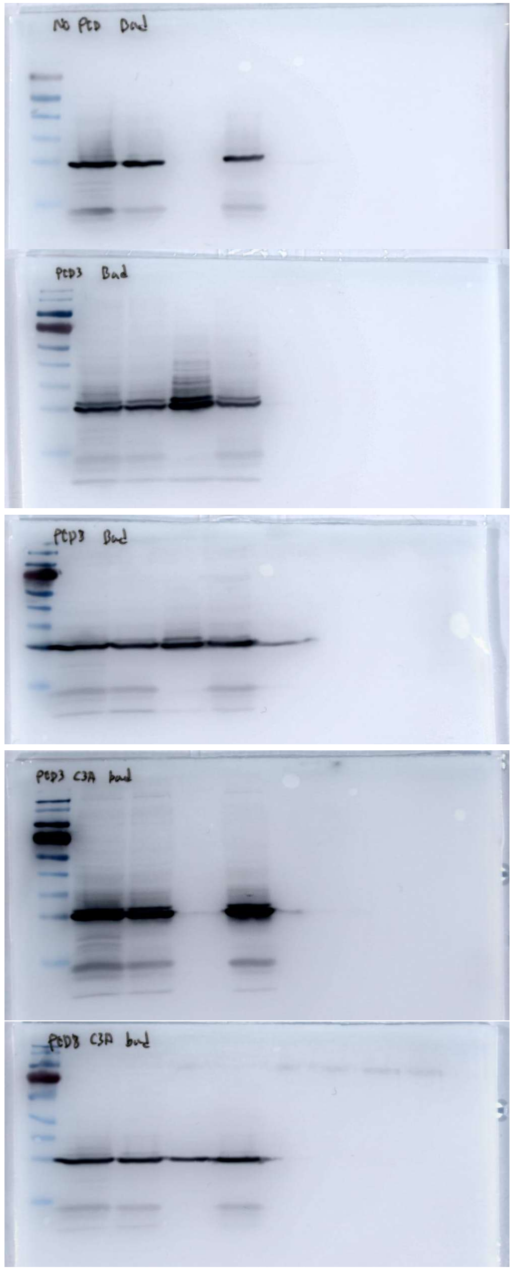

Figure.4D [2]

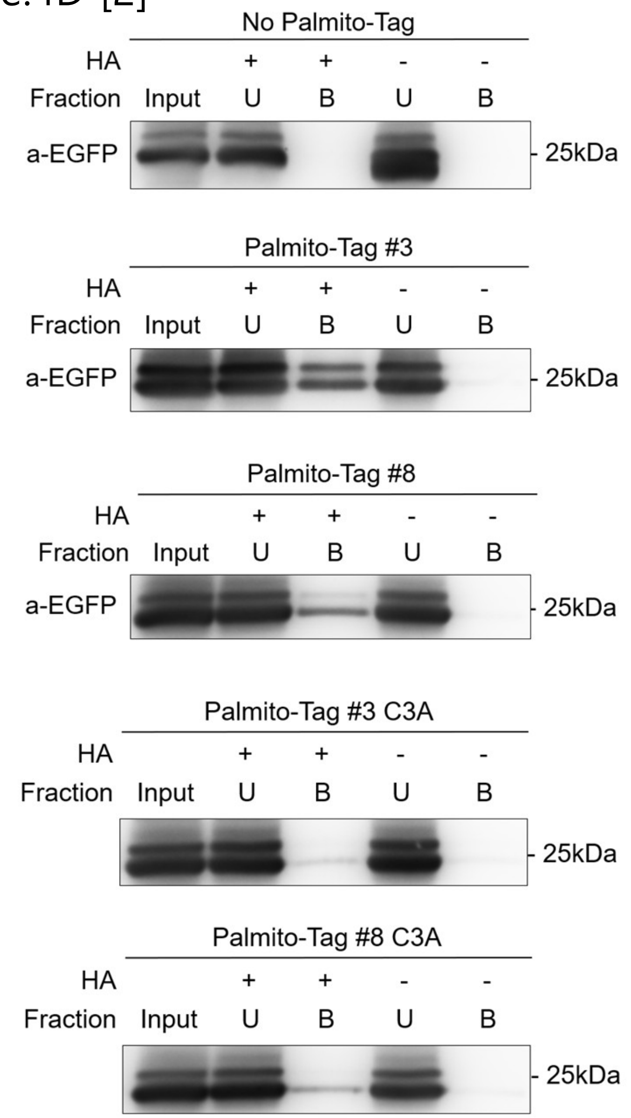
No Palmito-Tag
HA + + - -
Fraction Input U B U B
a-EGFP
25kDa
Palmito-Tag #3
HA + + - -
Fraction Input U B U B
a-EGFP
25kDa
Palmito-Tag #8
HA + + - -
Fraction Input U B U B
a-EGFP
25kDa
Palmito-Tag #3 C3A
HA + + - -
Fraction Input U B U B
25kDa
Palmito-Tag #8 C3A
HA + + - -
Fraction Input U B U B
25kDa

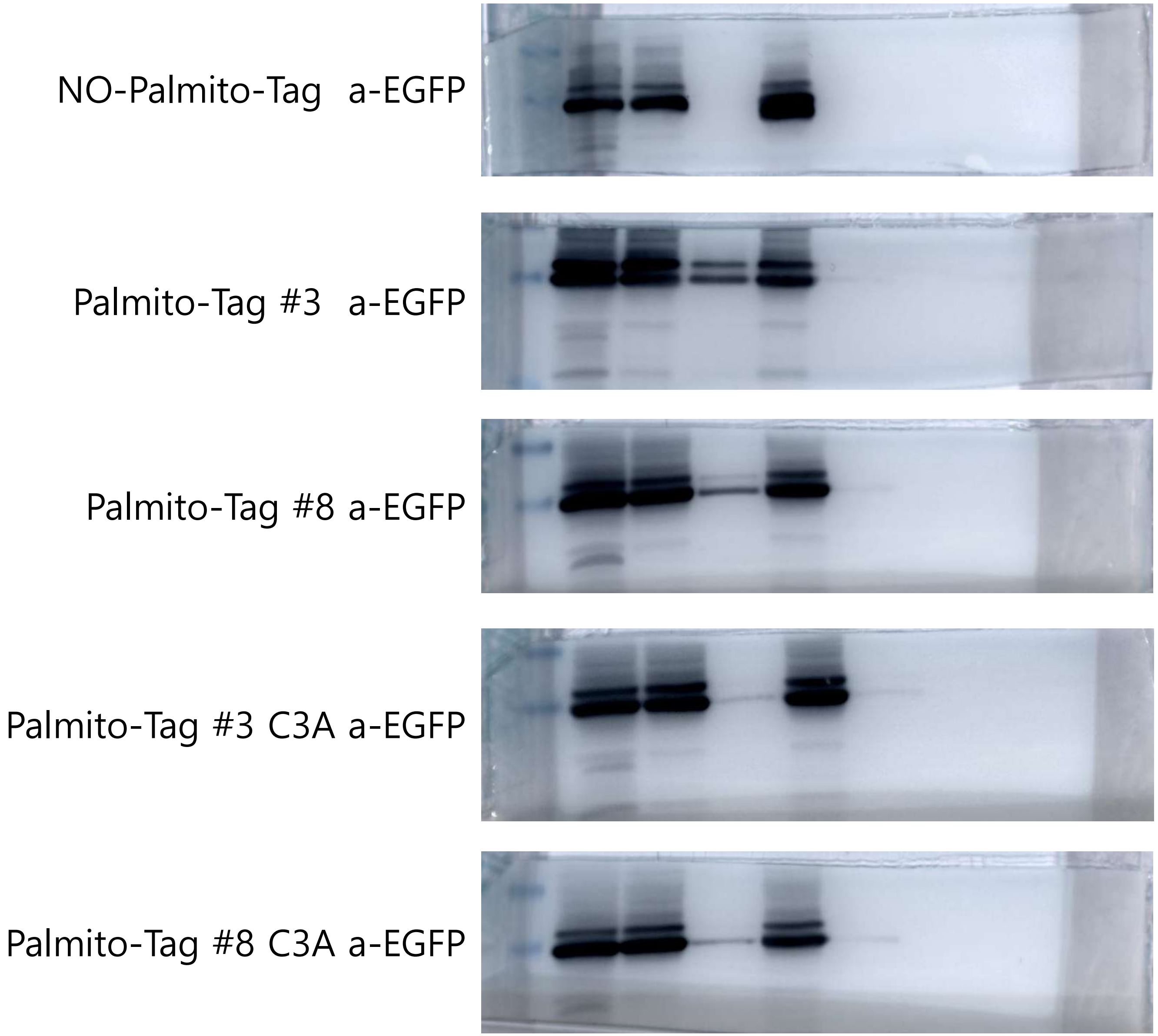
NO-Palmito-Tag a-EGFP
Palmito-Tag #3 a-EGFP
Palmito-Tag #8 a-EGFP
Palmito-Tag #3 C3A a-EGFP
Palmito-Tag #8 C3A a-EGFP

Figure.4D [3]

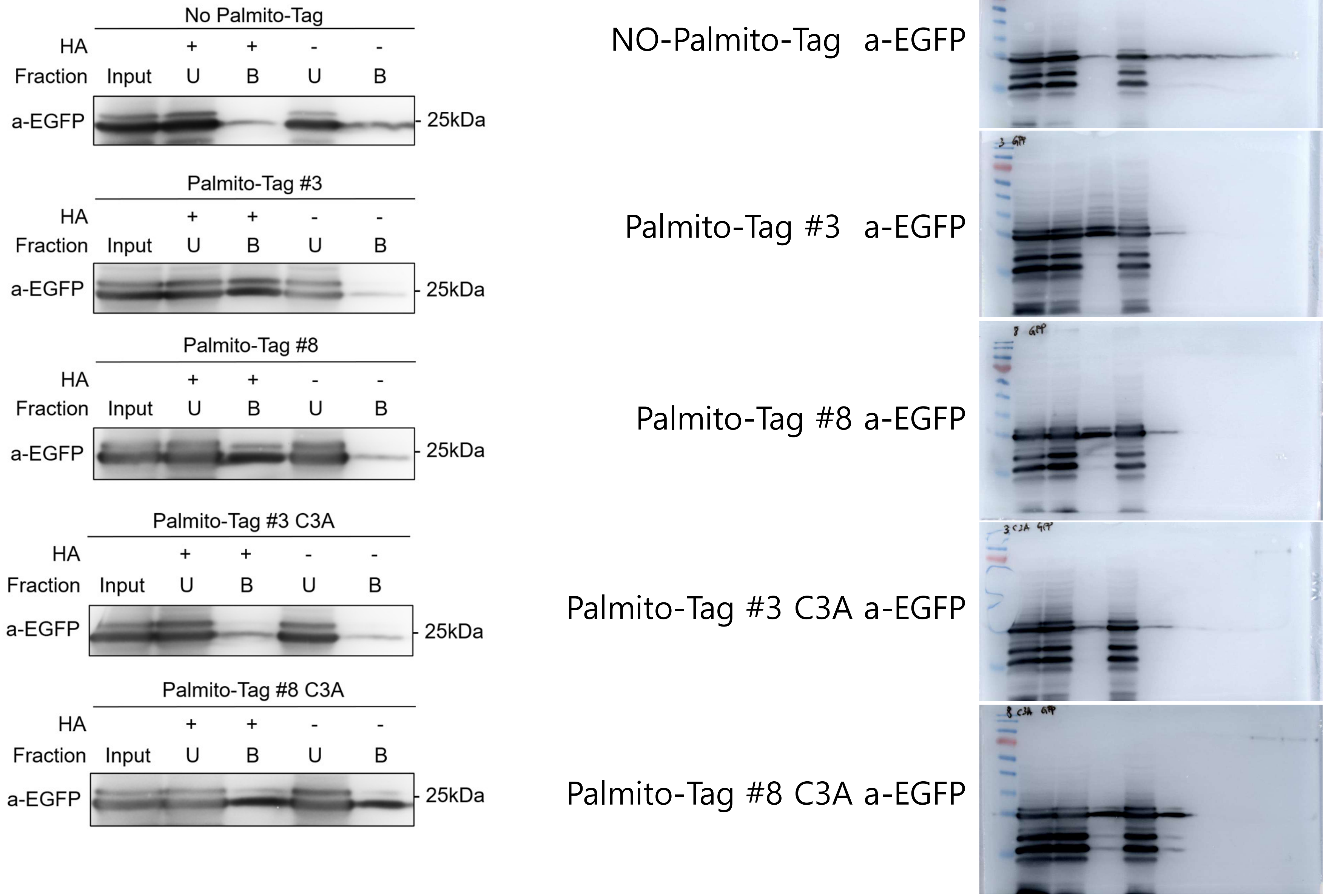
No Palmito-Tag
HA + + - -
Fraction Input U B U B
a-EGFP
25kDa
Palmito-Tag #3
HA + + - -
Fraction Input U B U B
a-EGFP
25kDa
Palmito-Tag #8
HA + + - -
Fraction Input U B U B
a-EGFP
25kDa
Palmito-Tag #3 C3A
HA + + - -
Fraction Input U B U B
a-EGFP
25kDa
Palmito-Tag #8 C3A
HA + + - -
Fraction Input U B U B
a-EGFP
25kDa
NO-Palmito-Tag a-EGFP
Palmito-Tag #3 a-EGFP
Palmito-Tag #8 a-EGFP
Palmito-Tag #3 C3A a-EGFP
Palmito-Tag #8 C3A a-EGFP

Figure.4E [1]

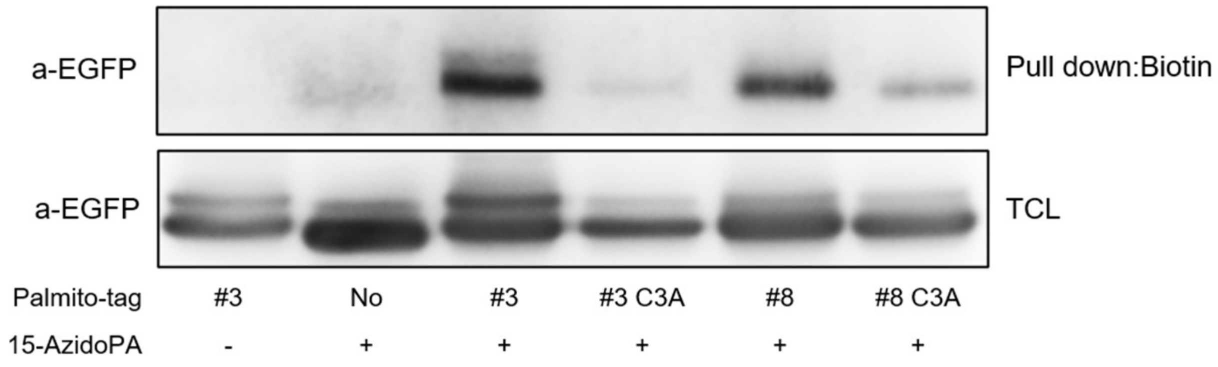

Pulldown-EGFP(25kDa)

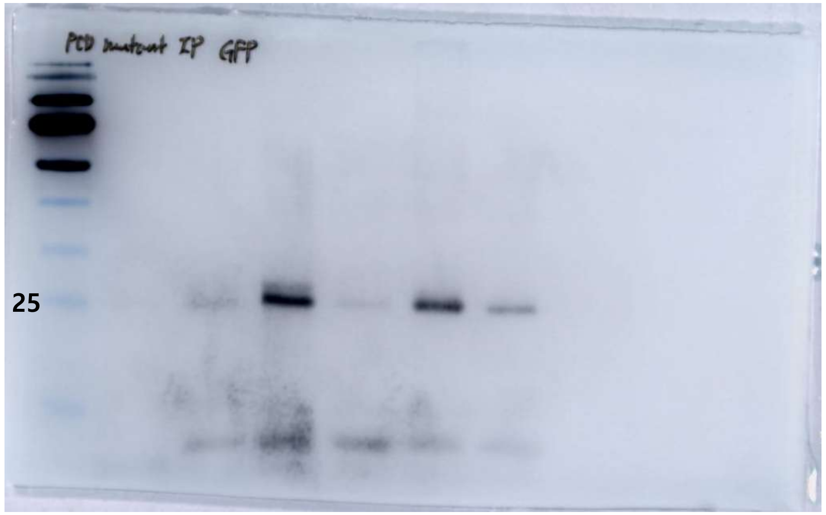

TCL-EGFP(25kDa)

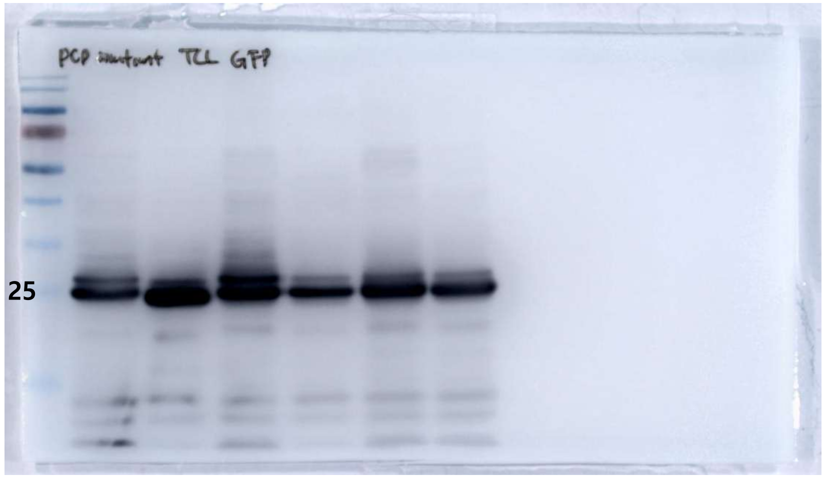

Figure.4E [2]

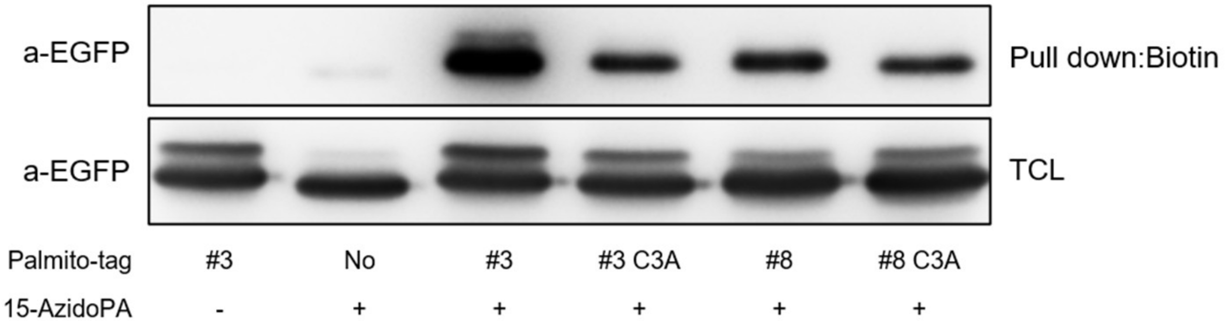

Pulldown-EGFP(25kDa)

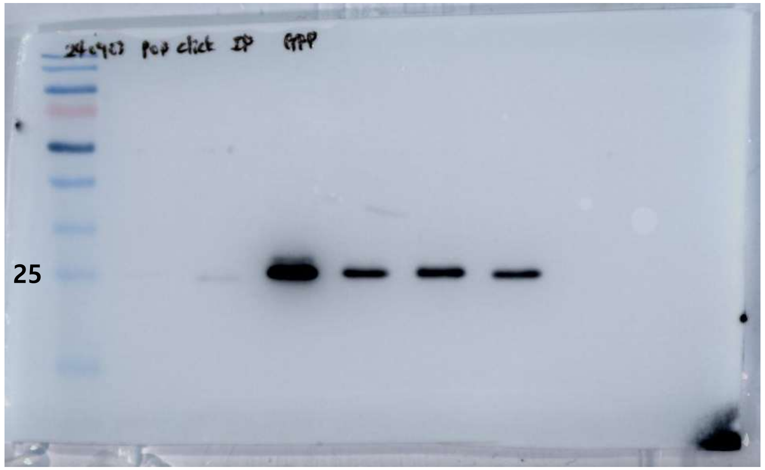

TCL-GFP(25kDa)

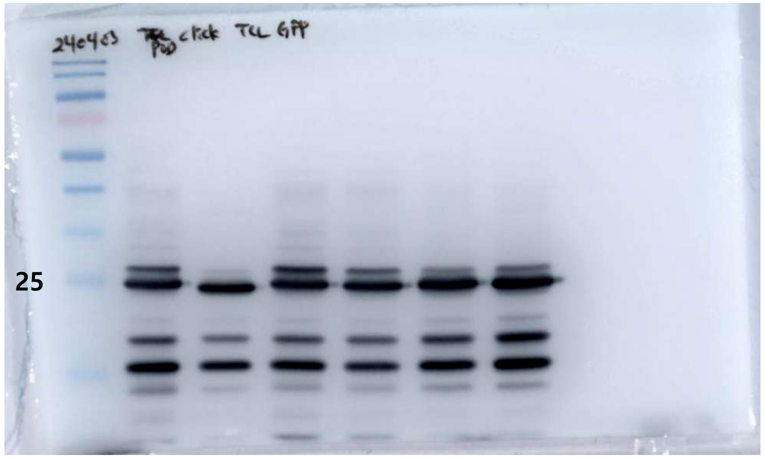

Figure.4E [3]

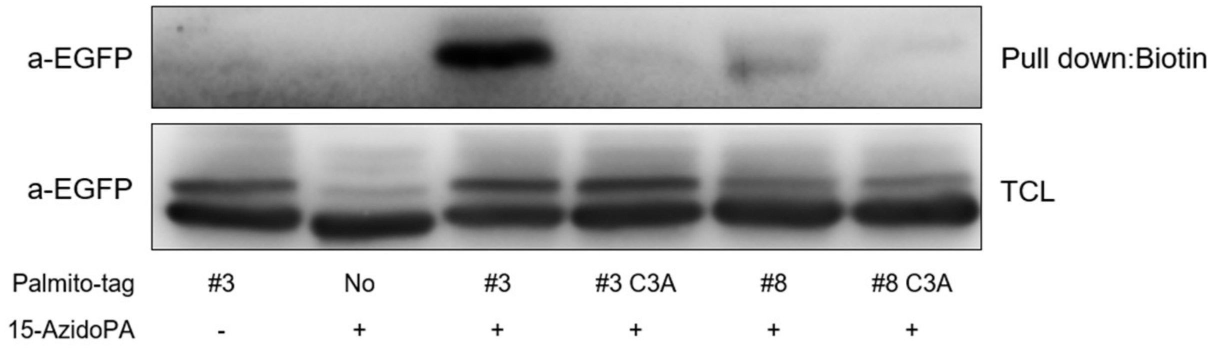

Pulldown-EGFP(25kDa)

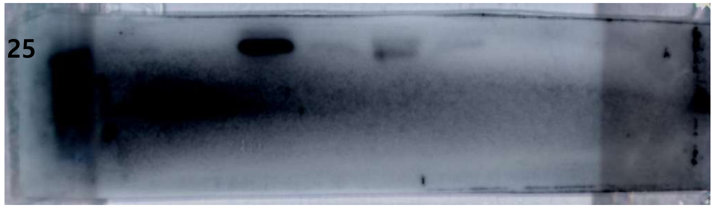

TCL-EGFP(25kDa)

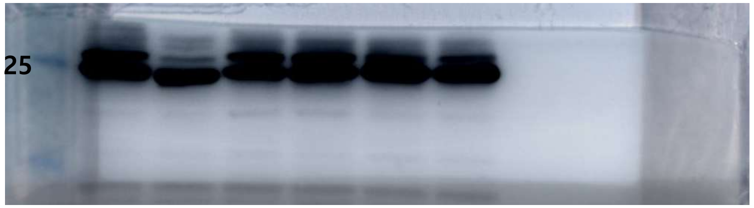

Supplement: Supplementary file 1 [file biomolecules-15-01076-s001.zip › original westerns.pdf]
